# Analyzing the soybean transcriptome during autoregulation of mycorrhization identifies the transcription factors GmNF-YA1a/b as positive regulators of arbuscular mycorrhization

**DOI:** 10.1186/gb-2013-14-6-r62

**Published:** 2013-06-18

**Authors:** Sara Schaarschmidt, Peter M Gresshoff, Bettina Hause

**Affiliations:** 1 Leibniz Institute of Plant Biochemistry (IPB), Weinberg 3, 06120 Halle (Saale), Germany; 2 Humboldt-Universität zu Berlin, Faculty of Agriculture and Horticulture, Division Urban Plant Ecophysiology, Lentzeallee 55-57, 14195 Berlin, Germany; 3 ARC Centre of Excellence for Integrative Legume Research (CILR), The University of Queensland, St. Lucia, Queensland 4072, Australia

**Keywords:** Affymetrix annexin, GeneChip, autoregulation, arbuscular mycorrhiza, CCAAT-binding transcription factor NF-Y, *Rhizophagus irregularis*, *Glycine max *(soybean), quantitative RT-PCR, split-root system

## Abstract

**Background:**

Similarly to the legume-rhizobia symbiosis, the arbuscular mycorrhiza interaction is controlled by autoregulation representing a feedback inhibition involving the CLAVATA1-like receptor kinase NARK in shoots. However, little is known about signals and targets down-stream of NARK. To find NARK-related transcriptional changes in mycorrhizal soybean (*Glycine max*) plants, we analyzed wild-type and two *nark *mutant lines interacting with the arbuscular mycorrhiza fungus *Rhizophagus irregularis*.

**Results:**

Affymetrix GeneChip analysis of non-inoculated and partially inoculated plants in a split-root system identified genes with potential regulation by arbuscular mycorrhiza or NARK. Most transcriptional changes occur locally during arbuscular mycorrhiza symbiosis and independently of NARK. RT-qPCR analysis verified nine genes as NARK-dependently regulated. Most of them have lower expression in roots or shoots of wild type compared to *nark *mutants, including genes encoding the receptor kinase GmSIK1, proteins with putative function as ornithine acetyl transferase, and a DEAD box RNA helicase. A predicted *annexin *named *GmAnnx1a *is differentially regulated by NARK and arbuscular mycorrhiza in distinct plant organs. Two putative *CCAAT-binding transcription factor *genes named *GmNF-YA1a *and *GmNF-YA1b *are down-regulated NARK-dependently in non-infected roots of mycorrhizal wild-type plants and functional gene analysis confirmed a positive role for these genes in the development of an arbuscular mycorrhiza symbiosis.

**Conclusions:**

Our results indicate *GmNF-YA1a/b *as positive regulators in arbuscular mycorrhiza establishment, whose expression is down-regulated by NARK in the autoregulated root tissue thereby diminishing subsequent infections. Genes regulated independently of arbuscular mycorrhization by NARK support an additional function of NARK in symbioses-independent mechanisms.

## Background

Plants have a long success story in hosting microsymbionts in their roots to improve their supply with mineral nutrients, particularly the two important macronutrients phosphate and nitrogen. The arbuscular mycorrhiza (AM) symbiosis, an interaction of plants with fungi of the phylum *Glomeromycota *[1], probably co-evolved with the early land plants around 450 million years ago (for overview see [2]). Nowadays, the majority of land plants can form an AM symbiosis that is characterized by the exchange of phosphate against monosaccharides [3]. Moreover, mycorrhizal plants can also benefit from improved availability of other minerals and water, and from induced abiotic and biotic stress tolerance, all contributing to higher plant biodiversity and productivity of ecosystems [4]. AM fungi are obligate biotrophs colonizing the root cortex of plants where they can form, depending on the plant and fungal species, inter- and intracellular hyphae, highly branched intracellular hyphae called arbuscules and/or intracellular hyphal coils, and vesicles that serve as storage organs (for overview see [5]).

In addition to the wide-spread AM interaction, a few plant families including leguminous and actinorhizal plants have evolved the capability to interact with nitrogen-fixing bacteria like rhizobia and *Frankia*, respectively. The bacterial symbionts are hosted intracellularly in specialized organs called root nodules. Studies on plant genes involved in the establishment of these intracellular root-microbe symbioses indicated a common evolutionary origin. This led to the assumption that genes involved in the AM symbiosis might have been recruited to allow the interaction with nitrogen-fixing bacteria (for review see [6-9]).

In leguminous plants, a whole set of genes are known to be essential to successfully establish the AM symbiosis and the legume-rhizobia symbiosis, referred to here as nodulation (for review see [7-10]). Establishment of both endosymbioses is initiated by an intense signal exchange between the partners. Nod and Myc factors contain lipochitooligosaccharide signals that are perceived by plant receptor kinases (RKs): in the case of nodulation by LysM RK(s) and in the case of an AM symbiosis by a still unknown receptor, which somehow interacts with the Nod factor receptor complex [11]. The signals are further processed by an early common signaling pathway including a plasma membrane-bound leucine rich repeat (LRR) RK that was found to be also essential for actinorhiza formation (for review see [7]). Activation of nuclear cation channels and induction of nucleoporin(-related) proteins are involved in generating specific oscillations of the Ca^2+ ^concentrations in the nucleoplasm and perinuclear cytoplasm in both, AM and nodulation. In both symbioses, the Ca^2+ ^signal is translated by a Ca^2+ ^calmodulin-dependent protein kinase (CCaMK) that activates in interaction with other proteins specific transcription factors (TFs) (for overview see [8-10]).

To reduce carbon losses and to maintain the mutualistic character of the symbioses, the plant tightly controls the infection with both heterotrophic endosymbionts. One common regulatory mechanism that limits the number of successful infection events is called autoregulation (autoregulation of nodulation (AON); autoregulation of mycorrhization (AOM)) (for review see [12-16]). The general autoregulation system of legumes comprises systemic and long-distance feedback inhibition initiated by early signals of the plant-microbe interaction suppressing subsequent infections. The key signal mediator of autoregulation is a CLAVATA1 (CLV1)-like RK, hereafter referred to as NARK (Nodulation Autoregulation Receptor Kinase) [17]. Grafting and split-root experiments revealed that NARK acts in the shoot, limiting infections systemically in the entire root system [18,19]. Mutant plants with defective NARK are characterized by a supernodulating phenotype and a nitrate- and acid-tolerant nodulation [20,21] and can also exhibit increased mycorrhizal colonization and higher arbuscule abundance [22-25]. However, in the AM symbiosis, the effect of autoregulation in the wild-type is often less obvious, because the fungus can spread longitudinally within the root cortex without forming new infections once it has entered the root. Nevertheless, the autoregulation effect becomes apparent in split-root plants in which colonization caused by previous and subsequent infections can be distinguished [26,27].

Nod factor application and cross-infections with rhizobia and AM fungi demonstrated initiation of the general autoregulation system by common early signals [28]. Corresponding to the *Arabidopsis thaliana *CLV system, CLE peptides are suggested as root-derived signals activating NARK in the shoot (for review see [14,15]). However, AM-induced CLEs are so far not described. The shoot-derived inhibitor (SDI), acting downstream of NARK in AON, has been characterized biochemically as a heat-stable, ethanol-soluble, low-molecular weight molecule which is unlikely an RNA or protein [29,30]. A putative receptor of SDI in the root might be encoded by *TML *[31]. NARK is described to affect phytohormone balances including reduction of the shoot-to-root auxin transport and of jasmonic acid biosynthesis in the shoot [32-34]. Recently, it was shown that NARK also affects the ubiquitin-dependent protein degradation pathway via regulation of *GmUFD1a *[35].

Although AON and AOM share common elements, not all *nark *mutants characterized by supernodulation exhibit an equivalent lack of AOM [36]. This indicates the existence of additional factors that are specific in controlling the establishment of both endosymbioses. In the present study, transcriptional changes occurring during mycorrhization in soybean (*Glycine max*) plants were investigated. To find NARK-regulated genes involved in AOM, we performed Affymetrix GeneChip analyses with split-root plants of Bragg wild-type and *nark *mutants partially inoculated with the AM fungus *Rhizophagus irregularis*. Selected NARK-regulated, AM-dependent and AM-independent, genes were verified using RT-qPCR. Among these genes, we identified two putative *CCAAT-binding TF subunits*, named *GmNF-YA1a *and *GmNF-YA1b*, that were downregulated by NARK in an AM-dependent manner. The CCAAT sequence is one of the most common *cis*-acting elements and was found in approximately 30% of 502 eukaryotic promoters [37] (for overview see [38-40]). In higher eukaryotes, CCAAT-boxes are found in all types of promoters, including constitutively expressed and inducible promoters. Proteins that bind to the CCAAT-box or to the inverted box have been characterized *inter alia *for mammals, yeast, filamentous fungi, and plants, and include proteins of the nuclear factor Y (NF-Y) family. DNA binding activities of NF-Ys, which are also called CBFs (CCAAT-binding factors) or HAPs (heme activating proteins), require a high conservation of the five nucleotides. NF-Ys usually act as heterotrimers. Two of the subunits (NF-YB, NF-YC/CBF-A, CBF-C/HAP3, HAP5) form a stable heterodimer, which can then interact with the third subunit (NF-YA/CBF-B/HAP2) allowing binding to the promoter region. In case of yeast, an additional acid subunit, HAP4, activates the HAP2,3,5 complex. In plants, NF-Ys can function in diverse processes (for overview see [41,42]) and a few NF-Y subunits have previously found to be upregulated during root endosymbioses [43-53]. Though, no NF-YA subunit that is involved in AM symbiosis or suppressed during root symbioses has been described so far.

## Results and discussion

### Experimental setup and mycorrhization phenotype of wild-type and *nark *mutant plants

**Figure 1:**
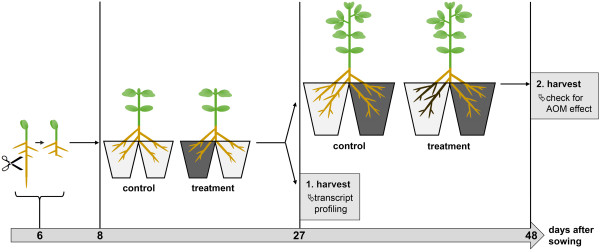
**Setup of split-root experiments**. Seeds of soybean wild-type, nts382, and nts1007 were germinated for 6 days. After cutting off the main root followed by 2 days of recovery, plants were transferred to the split-root system by dividing lateral roots on two pots. In doing so, one root-part of mycorrhizal plants ('treatment') was inoculated with *R. irregularis *(indicated by the dark color); the other root-part stayed non-infected. Control plants remained non-inoculated on both sides. Nineteen days after initial inoculation, part of the plants was harvested for transcript profiling and RT-qPCR analysis (1. harvest). Remaining plants were transferred to bigger pots to inoculate them with *R. irregularis *at another root-part to check for the AOM effect after another 21 days. In addition, in split-root experiments II and III, some of the initially inoculated plants were, without any subsequent inoculation, harvested at approximately 6 weeks after inoculation for further gene expression analysis (not shown). For pictures of plants see Figure S1 in Additional file 1.

**Figure 2:**
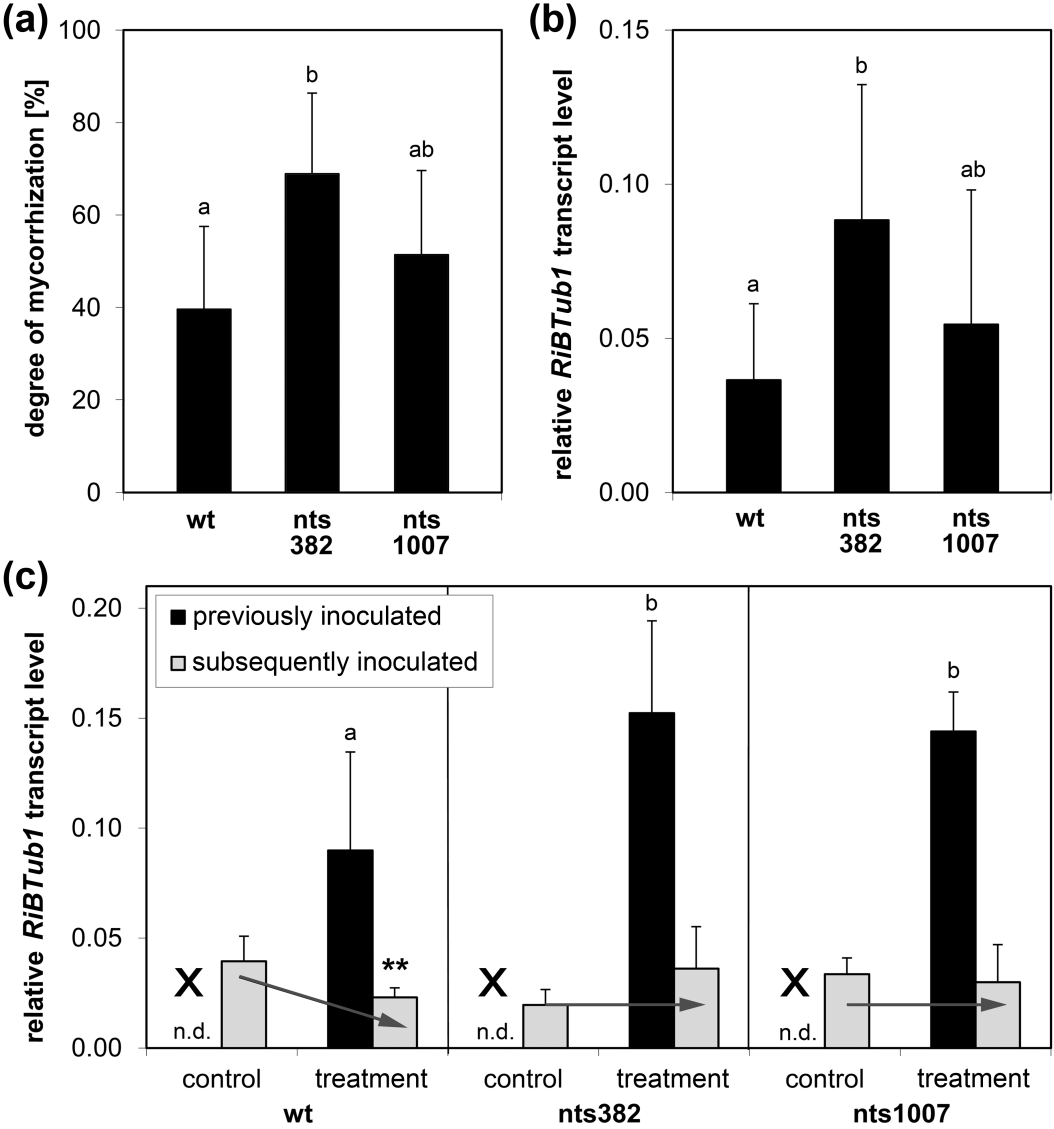
**Mycorrhization phenotype of wild-type, nts382, and nts1007 in the split-root experiment I used for Affymetrix GeneChip analysis**. (**a**, **b**) Colonization of initially inoculated root-parts at time-point of first harvest (= 19 days after inoculation with *R. irregularis*). (**c**) Colonization of both root-parts at time-point of second harvest (= 40 days after previous and 21 days after subsequent inoculation with *R. irregularis*) in either pre-inoculated ('treatment') or non-pre-inoculated ('control') plants. Crosses indicate the missing previous inoculation in case of control plants. The colonization of root-parts by *R. irregularis *was analyzed microscopically after staining of roots (a) and/or by transcript analysis of the fungal marker gene *RiBTub1 *(b, c). Transcript levels of *RiBTub1 *were determined by RT-qPCR and calculated in relation to *GmSUBI-1 *as plant reference gene. All data are given as mean values of six to seven plants + SD. Data of previously inoculated root-parts (a-c) were statistically analyzed by multiple Student's t-tests with Bonferroni correction. Different letters designate significant differences with *P *≤0.05. Data of subsequently inoculated root-parts (c) were pairwise compared between control plants and mycorrhizal plants ('treatment'), each for wild-type (wt), nts382, and nts1007, by the Student's t-test. **P ≤0.01. n.d.: not detected.

In general, a root system successfully inoculated with an AM fungus consists of colonized as well as non-colonized, but autoregulated roots. To physically separate autoregulated roots from roots containing fungal structures allowing transcript analysis of specifically regulated genes, we used a split-root system (for details see Materials and methods, Figure 1, and Figure S1 in Additional file 1). In the split-root system, one half of the root system of wild-type and *nark *mutant plants was inoculated with *R. irregularis*, while the other half remained non-inoculated. Corresponding controls stayed completely non-inoculated. In contrast to the AON, which is activated very rapidly after inoculation, previous studies indicate that activation of AOM requires a certain level of fungal colonization [54]. Thus, plants for Affymetrix analysis were harvested 19 days after inoculation. The colonization pattern was determined microscopically in cross-sections of root-parts and by fungal transcript analysis in the entire tissue of the root-parts. For the latter, we used the fungal *β-Tubulin *gene *RiBTub1 *[55], which can act as marker for viable fungal structures [56]. Nineteen days after inoculation, the degree of mycorrhization was around 40%, 69%, and 51% in wild-type, nts382, and nts1007 mutant plants, respectively (Figure 2a). Inoculated nts382 root-parts contained significantly more AM fungal structures than corresponding wild-type root-parts. The transcript accumulation of the fungal marker gene *RiBTub1 *in the entire root-part tissue (Figure 2b) correlated well with the degree of mycorrhization analyzed microscopically in cross-sections taken out of the root-parts. Thus, *RiBTub1 *transcript accumulation was subsequently used to quantify fungal colonization.

To ensure that the autoregulation system was activated at the time-point of first harvest, some of the plants were then inoculated at the second root-part and mycorrhization was analyzed after another 3 weeks. At this time-point, the mRNA level of *RiBTub1 *in the initially inoculated root-parts was significantly higher in both *nark *mutants compared to the wild-type (Figure 2c; 'previously inoculated'). No *RiBTub1 *transcript accumulation was detected in corresponding root-parts of control plants. In the later inoculated root-parts, the *RiBTub1 *mRNA level indicates that subsequent infections led to approximately 40% lower colonization rates in wild-type plants previously inoculated at the other root-part compared to wild-type plants without previous infection (Figure 2c; 'subsequently inoculated'). In contrast, both *nark *mutants, nts382 and nts1007, showed a defective autoregulation. Here, the *RiBTub1 *transcript level was not affected by previous inoculation. For validation of NARK-regulated gene expression, the experiment was repeated twice (split-root experiment II and III) reflecting the mycorrhization phenotypes and the AOM effect found in the split-root experiment I (Figure S2 in Additional file 1).

For the *nark *mutant nts1007 a missing autoregulation has been previously described for nodulation as well as for mycorrhization [17,22]. This mutant has a nonsense mutation that truncates the RK protein at glutamine residue 106 (Q106*) eliminating most of the LRRs and the entire kinase domain [17]. In contrast, the nts382 mutant (Q920*) has a nonsense mutation in the C-terminal part of the kinase domain [17] and might still exhibit some kinase activity. Previous studies on the *En6500 *mutant (K606*) and some other uncharacterized *nark *mutants of the soybean cv. Enrei indicated a lacking AOM in these supernodulating mutants, suggesting that a lower NARK activity is sufficient to trigger AOM than AON [36]. However, in the present study, we found the Bragg *nark *mutant nts382 to be, in addition to the defective AON [17], also severely affected in AOM (Figure 2, Figure S2 in Additional file 1). Because in nts382 the truncated NARK contains even more of the kinase domain than in *En6500*, differences in AOM between *nark *mutants of Bragg and of Enrei might somehow be rather cultivar-related.

Because *nark *mutants are characterized by a nitrogen tolerant nodulation, we additionally analyzed the mycorrhization phenotype under different phosphate and nitrogen supply conditions (Figure S3 in Additional file 1). In wild-type plants, AM fungal colonization was suppressed by increasing fertilization with phosphate. In addition, both *nark *mutants showed a decrease in mycorrhizal colonization in response to increasing levels of phosphate (Figure S3a in Additional file 1). Under the tested conditions, however, a suppression of the mycorrhizal colonization by nitrogen was not found - neither in *nark *mutants nor in the wild-type (Figure S3b in Additional file 1). The lower mycorrhization under strong nitrate limitation might be caused by reduced carbon supply of the fungus as plants showed chlorosis and growth reduction (Figure S3c in Additional file 1).

### Analyzing the soybean transcriptome during AOM by the Affymetrix GeneChip

Transcript profiling using the Affymetrix GeneChip for soybean was conducted with material of Bragg wild-type and of the *nark *mutant nts1007, both genotypes either non-inoculated or partially inoculated with *R. irregularis*. The *nark *mutant nts1007 was previously shown to be defective in AOM [22] and used for Affymetrix analysis of nodulated seedlings [32]. To find genes regulated by AM and NARK, the Affymetrix data were screened by (multiple) pairwise comparisons using the dChip software [57] as described in the Materials and methods section.

**Figure 3:**
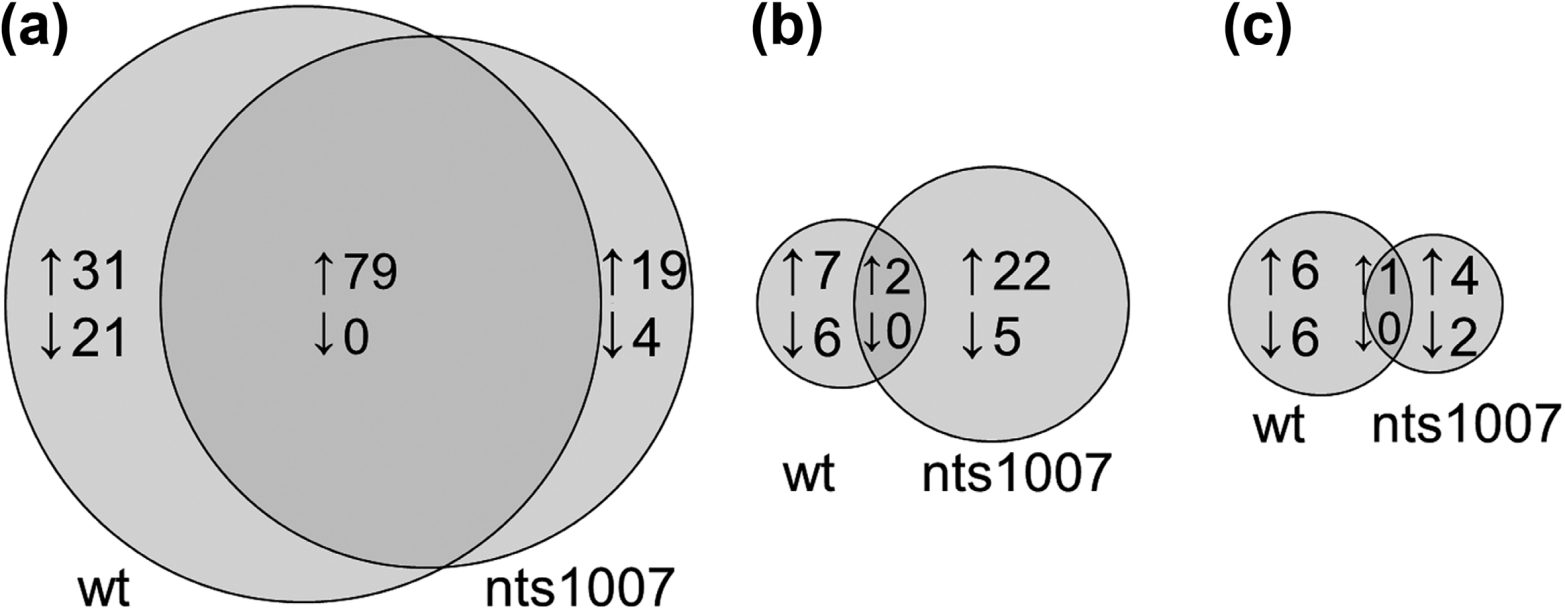
**Effect of *R***. *irregularis *on local and systemic gene expression in wild-type and nts1007. (**a**) Number of genes regulated locally by *R. irregularis *in wild-type (wt) and nts1007 roots. (**b**, **c**) Number of genes regulated systemically by *R. irregularis *in shoots (b) and non-inoculated root-parts (c) of wild-type and nts1007 plants. Affymetrix gene expression analysis was done for plants of the split-root experiment I harvested 19 days after initial inoculation (see Figure 1). Left circles and right cycles indicate AM-regulated genes in wild-type and nts1007, respectively, whereas overlaying areas indicate genes that fulfill the criteria in both genotypes. Criteria for all comparisons were >2-fold changes with *P *≤0.1 for all unpaired and paired t-tests performed with dChip (*n *= 3). ↑: upregulated genes ↓: downregulated genes.

The colonization of wild-type and nts1007 roots with *R. irregularis *resulted in a local, more than two-fold upregulation of 110 and 98 genes, respectively (Figure 3a). Few genes were found to be downregulated in mycorrhizal wild-type or nts1007 roots. Most of the locally induced genes seemed to be regulated independent of NARK because 79 of them were found to be upregulated in mycorrhizal wild-type and *nark *mutant roots. Twenty-five genes were >5-fold and eight genes were between 20- to 200-fold higher in mycorrhizal roots of wild-type and nts1007 plants compared to non-inoculated roots. Most of the locally AM-induced genes are putatively involved in metabolism (24 genes common in both genotypes) and secondary metabolism (11 genes); the others are predicted to belong to eight more categories (for further information see Table S1 in Additional file 2). The upregulation of a whole set of genes of diverse functional categories is consistent with previous gene expression studies of mycorrhizal roots of other leguminous and non-leguminous plant species, such as *Medicago truncatula *[58-60], *Lotus japonicus *[61-63], or rice [64], reflecting the strong reprogramming of root tissue interacting with AM fungi.

In contrast to the strong changes in gene expression of mycorrhizal root tissue and to a previous analysis on systemic changes in mycorrhizal *M. truncatula *[63], only some soybean genes were found to show a systemic AM-response in shoots and/or non-colonized root-parts of mycorrhizal plants under the given criteria (Figures 3b and 3c). The overall expression patterns of most of these genes, however, hardly indicate a putative regulation by NARK (data not shown). Thus, to find additional candidates potentially regulated by NARK, the criteria of pairwise comparisons were weakened and putative NARK-regulated genes controlled in a rather AM-independent manner were included. The Affymetrix data were screened again by performing multiple pairwise comparisons with dChip and by using the clustering software CLANS [65]. A distinct gene cluster reflecting a putative regulation by NARK was not found by CLANS. However, using CLANS to screen the genes for a specific expression pattern, we found mostly the same genes as found by the multiple pairwise comparisons with dChip. After screening, candidates for further analysis were selected manually due to their overall expression pattern within all samples that indicate a putative AM-dependent or AM-independent regulation by NARK (further information is provided in Table S2 in Additional file 2). Among these candidates, several genes were identified with a putative function as early nodulin, TF, zinc finger protein, cytochrome P450, calmodulin, pathogenesis-related protein, and with transport function including a putative upregulated amino acid transporter (Glyma19g22590).

### NARK-regulated candidate genes validated by RT-qPCR

For a first validation of the selected candidates, we used other plants of the first experiment (split-root experiment I) than those used for the Affymetrix analysis. Candidates showing different mRNA levels in shoots and/or non-colonized root-parts of inoculated wild-type compared to nts1007 plants were analyzed in more detail (Table S2 in Additional file 2 see also Figure S4 in Additional file 1). If the expression pattern in all treatments - including nts382 and non-inoculated control plants - indicated a regulation by NARK, gene expression was validated in plant material of at least two independent experiments in total. Nine genes were found to be differently expressed (with *P *≤0.05) in wild-type and both *nark *mutants (Table 1). For several others, including, for example, predicted *NAC TFs*, *transporters*, and a gene involved in abscisic acid biosynthesis, qRTâ€�PCR data might point to a NARKâ€�regulation (Table S2 in Additional file 2). However, further analyses are required to support such a regulation for these genes. One of the nine verified genes, Glyma15g15171, that encodes a protein of unknown function, had higher transcript levels in the shoots of wild-type plants than in *nark *mutants (Figure S5a in Additional file 1). For all others, mRNA levels were lower in wild-type than in *nark *mutants; mostly in roots or in both, roots and shoots. NARK regulation was also found at 40 days after inoculation (Figures S6a-f in Additional file 1). Most of the validated genes did not show significant AM-dependency of NARK regulation; one putative *annexin *was AM-induced in roots but NARK-suppressed in shoots, and two putative *TFs *were identified as NARK-regulated in an AM-dependent manner (see below).

**Table 1:** Validated NARK regulation of soybean genes in *R.*

Gene locus	Affymetrix probe set(s)	Predicted gene function/Name	wt/*nark *(Affymetrix)^a^		wt/*nark*_¬ _(RT-qPCR)^b^	
				
			Shoots	Roots	Shoots	Roots
Glyma15g15171	GmaAffx.30002.1.S1_at	Unknown	**1.86**	0.80	**1.97**	n.a.

Glyma18g17440	Gma.7686.1.S1_at	Ornithine acetyl transferase	**0.17**	**0.12**	**0.028**	**0.0065**

Glyma02g11150 [UniGen:EU669879]	GmaAffx.82595.1.S1_at GmaAffx.82595.2.S1_at	Stress-induced receptor-like kinase *GmSIK1*	**0.22 0.30**	**0.02 **0.08	**0.25**	**0.23**

Glyma17g09270	GmaAffx.68580.1.S1_at GmaAffx.46141.1.S1_at	DEAD box RNA helicase	**0.71 0.74**	**0.65 0.71**	**0.54**	**0.60**

Glyma10g35000	Gma.6487.1.A1_at	Unknown	**1.37**	**0.44**	n.a.	**0.49**

Glyma07g36986	Gma.17992.1.S1_at	Unknown	**0.62**	**0.50**	**0.58**	**0.59**

Glyma15g38010	Gma.3440.2.S1_at Gma.3440.2.S1_a_at	Annexin/*GmAnn1a*	**0.37 0.43**	**0.82 **0.79	**0.30**	0.90

Glyma03g36140	GmaAffx.40657.1.S1_at	NF-YA/*GmNF-YA1a*	**0.70**	**0.56**	0.84	**0.63**

Glyma19g38800	GmaAffx.40657.1.S1_at	NF-YA/*GmNF-YA1b*	**0.70 **	**0.56**	0.76	**0.62**

*irregularis*-inoculated plants.

^a^The value gives the mean ratio between the mRNA accumulation in mycorrhizal plants of Bragg wild-type (wt) and of the *nark *mutant nts1007 19 days after inoculation determined by Affymetrix gene expression analysis (*n *= 3). Data for roots derived from non-inoculated root-parts of mycorrhizal plants. Bold numbers indicate values with *P *≤0.1 (Student's t-test). Details are provided in Table S1 in Additional file 2.

^b^The value gives the mean ratio between the mRNA accumulation in mycorrhizal plants of Bragg-wild type (wt) and of both *nark *mutants 19 days after inoculation determined by RT-qPCR analysis in biological replicates different to those used for Affymetrix GeneChip analysis (*n *≥8 of two to three independent experiments). Data for roots derived from non-inoculated root-parts of mycorrhizal plants. Bold numbers indicate values with *P *≤0.05 (Student's t-test). n.a.: not analyzed.

Symbioses-dependent control of gene expression by NARK might to some extent occur in specific cell-types and/or transiently only. Transient regulation could particularly take place in cases of induction or *de-novo *synthesis of gene products. A specific temporal- and/or spatial-restricted NARK regulation might also explain the low number of genes identified in our study. In contrast to a previous analysis of nodulated seedlings [32], no suppression of JA-biosynthesis or -response genes was found by AM or by NARK. This might be due to the different developmental stages of the plants analyzed. Even in the work by Kinkema and Gresshoff [32], the differences between non-inoculated wild-type and nts1007 were less pronounced with increasing age of plants, showing mostly no changes in 17-day-old plants. Such transiently and developmentally controlled changes might be less reflected in our 27-day-old soybean plants. However, whether the changes previously found in nodulated soybeans were not detectable in mycorrhizal plants because they are cell-type specific, transiently and/or developmentally regulated or because they are specific for nodulation remains unknown.

### NARK-regulated genes lacking obvious regulation by AM

**Figure 4:**
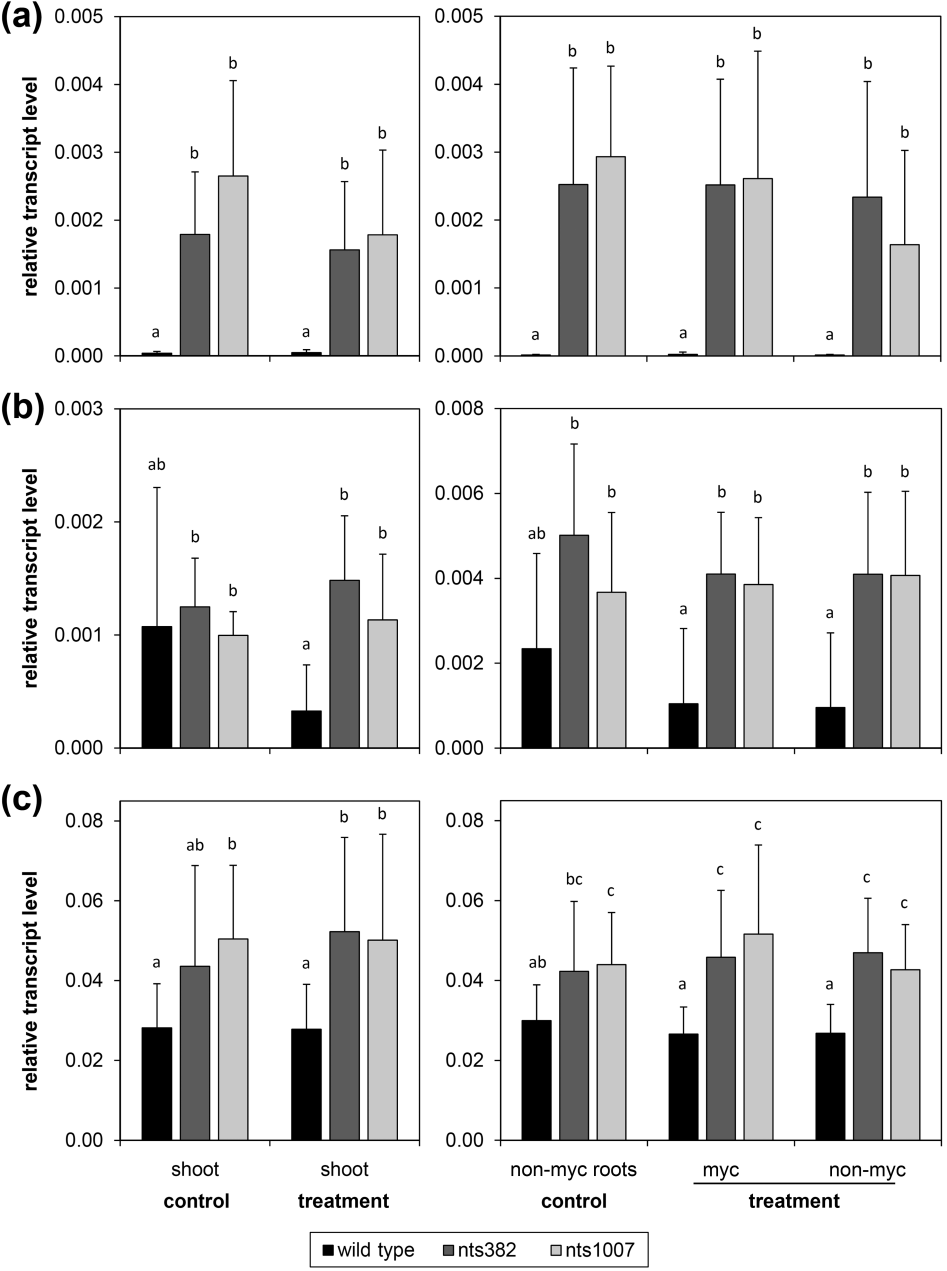
**Putative NARK-responsive genes in soybean plants 19 days after inoculation**. Transcript accumulation of all genes is shown for shoots (left) and root-parts (right) of non-inoculated ('control') and partially *R. irregularis*-inoculated plants ('treatment'). Gene expression in mycorrhizal plants was separately analyzed for the inoculated root-part ('myc') and for the non-inoculated, autoregulated root-part ('non-myc'). (**a**) Glyma18g17440, putative *ornithine acetyl transferase*. (**b**) Glyma02g11150, *Stress-induced receptor-like kinase 1 *(*GmSIK1*). (**c**) Glyma17g09270, putative *DEAD box RNA helicase*. Relative transcript levels were determined by RT-qPCR in wild-type and *nark *mutant (nts382 and nts1007) plants harvested 19 days after partial inoculation with *R. irregularis *(for details see Figure 1). Data are mean values + SD with *n *= 9-18 and *n *= 8-15 for wild-type and *nark *mutants, respectively, coming from at least two independent experiments. Different letters indicate significant differences (*P *≤0.05, multiple Student's t-tests with Bonferroni correction).

Using RT-qPCR and independent biological replicates not used for Affymetrix GeneChip analyses, we verified six genes as putative NARK-regulated in an AM-independent manner, which also did not show NARK-independent regulation by AM. Transcripts of one gene, Glyma18g17440, were almost not detectable in wild-type tissue but present in both *nark *mutants, independently on tissue type and/or *R. irregularis *inoculation (Figure 4a; Figures S6a,g in Additional file 1). Glyma18g17440 belongs to the ArgJ family and is putatively involved in arginine biosynthesis by encoding a protein with ornithine acetyl transferase (OAT) activity [GO:0004358]. OAT reversibly converts *N*^2^-acetyl-L-ornithine and L-glutamate into L-ornithine and *N*-acetyl-L-glutamate. Ornithine is a precursor of the synthesis of polyamines, such as putrescine (Put), spermidine (Spd), and spermine (Spm), and content of those metabolites was previously found to be altered in a supernodulating soybean mutant indicating repressed Spd and Spm biosynthesis from their precursor Put [66]. Interestingly, exogenously applied Spd and Spm can reduce the nodulation of the *nark *mutant [66] indicating a function in AON. It is tempting to speculate that the putative OAT might promote endosymbioses formation in *nark *mutants by affecting the availability of ornithine needed for polyamine synthesis. However, polyamines as well as ornithine and *N*^δ^-acetylornithine are described as general regulators of plant development and abiotic and biotic plant stress tolerance [67-69]. Thus, the considerable and constitutive differences in Glyma18g17440 mRNA accumulation between wild-type and *nark *mutants could also be involved in another NARK-controlled process not linked to the formation of root endosymbioses.

Transcript accumulation of the stress-induced receptor-like kinase *GmSIK1 *[UniGen:EU669879] in mycorrhizal plants was on average around four times lower in roots and shoots of wild-type compared to both *nark *mutants (Figure 4b). However, due to a strong variation in non-mycorrhizal wild-type, no significant AM dependency of NARK regulation was detected. Except from shoot tips that contain relatively high transcript levels, transcript accumulation of *GmSIK1 *increased with increasing age of above- and below-ground tissue (Figure S6h in Additional file 1), indicating a role of this abiotic stress-induced RK in processes linked to plant development or senescence.

According to our RT-qPCR data, transcript levels of the other putative NARK-suppressed genes were overall around 30% to 50% reduced in wild-type compared to *nark *mutant tissue. One of the genes that were rather slightly downregulated by NARK in roots and shoots is a predicted *DEAD box RNA helicase *(Glyma17g09270) (Figure 4c). Gene transcripts were highest in tips of shoots and roots (Figure S6j in Additional file 1). Glyma17g09270 is targeted by the two Affymetrix probe sets GmaAffx.68580.1.S1_at and GmaAffx.46141.1.S1_at. The latter, which as well reflects NARK regulation of target gene(s), might also detect the homologous gene Glyma05g02590. RT-qPCR analysis does not indicate gene regulation by NARK for this homolog (data not shown). DEAD box RNA helicases are widely occurring proteins with diverse activities including ATP binding and ATPase activity as well as RNA binding and unwinding or transport activity and are associated with nearly all processes that involve RNA, including development and stress responses [70,71]. Moreover, two genes of unknown function, Glyma10g35000 and Glyma07g36986, were downregulated by NARK in roots (Figures S5b, c in Additional file 1). Glyma07g36986 mRNA levels were also slightly lower in shoots of mycorrhizal wild-type compared to both *nark *mutants.

In general, AM-independent gene regulation by NARK indicates an additional role of NARK in other, symbioses-independent processes as previously been proposed [32]. Differences in polar auxin transport or JA biosynthesis are also found between non-inoculated *nark *mutant and wild-type plants [22,32-34]. This indicates that even non-inoculated plants exhibit a certain NARK activity. Indeed, symbioses-independent phenotypes of *nark *mutants of *L. japonicus *and *M. truncatula *[72,73] support a function of NARK in root growth and/or lateral root formation. Part of the NARK-regulated genes identified in the present study was characterized by a development-dependent expression pattern. Hence, such genes might be involved in NARK-controlled signaling in plant development and/or in plant stress responses not linked to AM formation. However, even genes lacking obvious AM dependency in NARK regulation could play a role in AM symbiosis and in AOM as indicated by gene expression data of the putative *annexin *Glyma15g38010.

### *GmAnn1a *as a differentially NARK- and AM-regulated *annexin*

**Figure 5:**
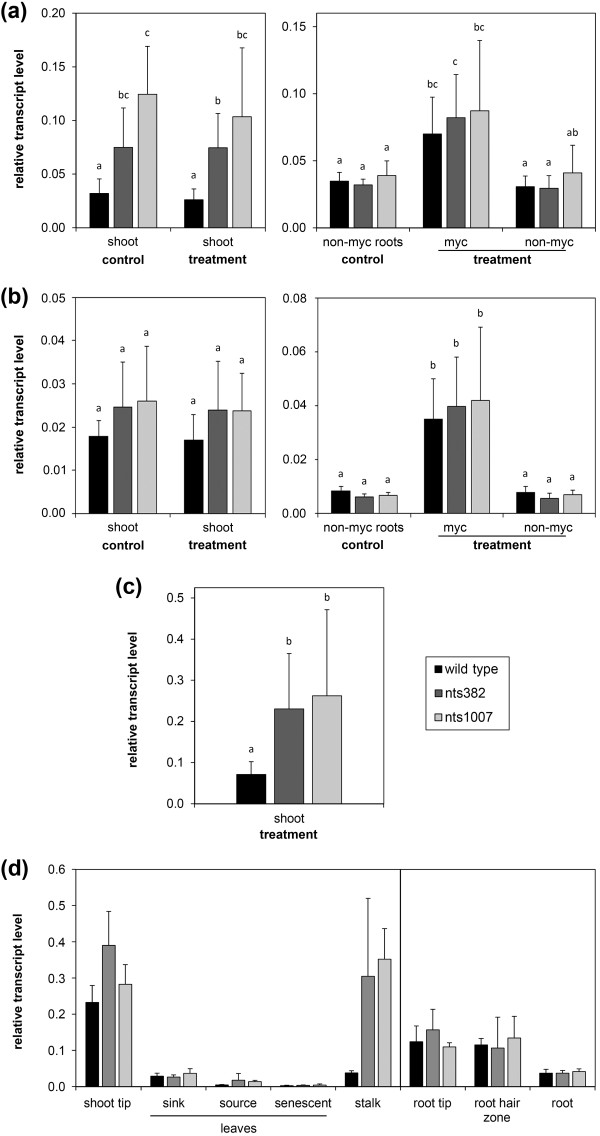
**Transcript accumulation pattern of two homologous *annexins *in wild-type and *nark *mutant plants**. (**a-c**) Relative transcript levels of Glyma15g38010, predicted *annexin *named *GmAnn1a *(a, c), and Glyma13g26960, predicted *annexin *named *GmAnn1b *(b), in split-root plants of wild-type and *nark *mutants (nts382 and nts1007). Plants were harvested 19 days (a, b) and 40 days (c) after partial inoculation with *R. irregularis *(for details see Figure 1 and legend of Figure 4). Data are means + SD with *n *≥10 and *n *≥8 for wild-type and nark mutants, respectively, coming from at least two independent experiments. Different letters indicate significant differences (*P *≤0.05, multiple Student's t-tests with Bonferroni correction). (**d**) Relative transcript levels of *GmAnn1a *in different tissues of non-inoculated 7-week-old wild-type and *nark *mutant (nts382 and nts1007) plants. Mean values + SD of plants of one experiment, wild-type: *n *= 5-6, nark mutants: *n *= 3. All transcript levels were determined by RT-qPCR and set in relation to *GmSUBI-1 *(a-d).

Here, we identified a putative soybean *annexin *gene named *GmAnn1a *(Glyma15g38010) that was locally upregulated by AM (independently of NARK) in roots but downregulated by NARK (independently of AM) in shoots (Figures 5a, c). When different plant tissues were compared, a higher transcript accumulation in stalks of *nark *mutants was detected compared to the wild-type (Figure 5d). In general, the putative *annexin *was predominantly expressed in young tissue, particularly in shoot and root tips and root hair zones. RT-qPCR analysis revealed that Glyma13g26960 (named *GmAnn1b*), the closest homolog of *GmAnn1a *and additional target of the Affymetrix probe set Gma.3440.2.S1_a_at, was as well locally induced by AM. However, *GmAnn1b *did not show significant regulation by NARK in shoots (Figure 5b). The Affymetrix data indicated that another predicted *annexin*, Glyma04g27100 targeted by GmaAffx.1082.1.S1_at, was induced by AM in mycorrhizal roots (Figure S7a in Additional file 1, see also Table S1 in Additional file 2). Its closest homolog, Glyma11g21457 targeted by GmaAffx.1082.1.A1_at, might be as well suppressed by NARK in shoots but was not induced by AM according to the Affymetrix data (Figure S7b in Additional file 1). Annexins bear membrane-associated functions that can be involved in vesicle transport and localized secretion, but may also act in Ca^2+ ^signaling, and they often have a key role in signal transduction and post-translational regulation in plant stress responses (for overview see [74,75]). In addition to the AM-induced soybean annexins identified in the present study, annexins of *M. truncatula *(MtAnn1/MtC20218, MtAnn2/MtC20219, MtC10763) were previously found to be transcriptionally and/or post-translationally upregulated in mycorrhizal root tissue and root nodules [59,76,77]. The MtAnn1 protein exhibits typical Ca^2+^-dependent binding to acid phospholipids and is supposed to be involved in early common signaling either in generating secondary messengers or by acting as Ca^2+ ^channel [75,77]. For MtAnn2 a function in cytoskeleton rearrangement or in the formation of the specialized host membrane surrounding endosymbionts has been suggested [59]. Thus, the AM-induced soybean annexins might act in a similar way. Additionally, AM-independent suppression of *GmAnn1a *by NARK probably occurred in the shoot vascular system indicating a role in long-distance signaling (Figure 5d). Indeed, annexins were found in the phloem, putatively involved in transport of phospholipids via the phloem [78]. Therefore, the fact that expression of *GmAnn1a *is suppressed in the stalk by NARK leads to the question whether it is connected to AOM, which will be subject of future studies.

### *GmNF-YA1a/b *as AM-dependently NARK-regulated *TF subunits*

**Figure 6:**
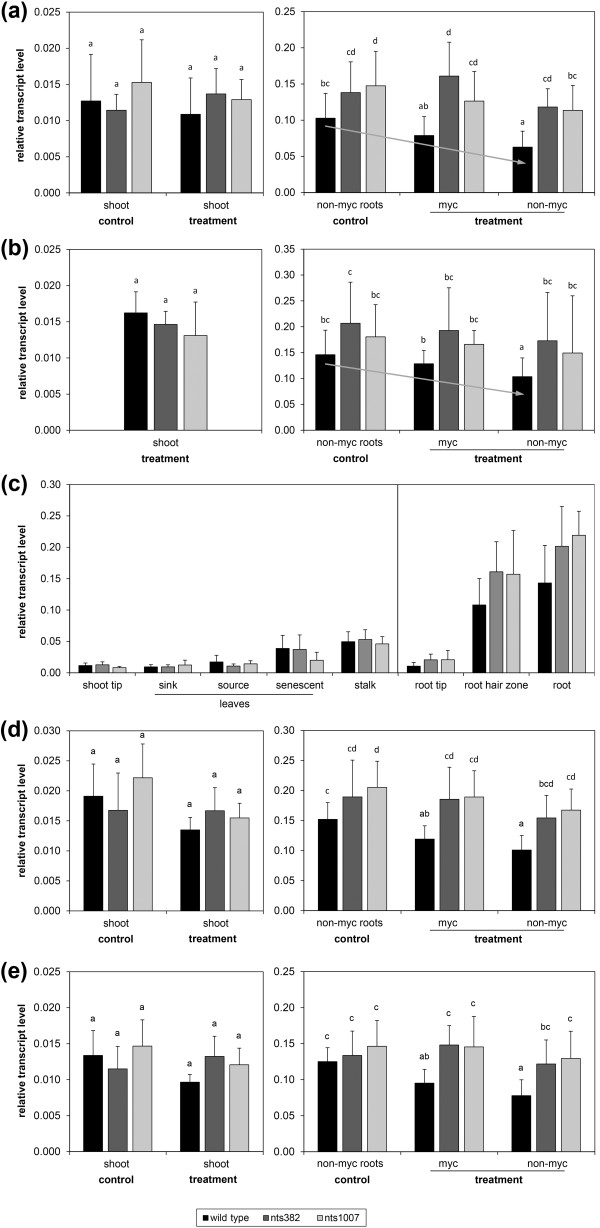
**Transcript accumulation pattern of *GmNF-YA1 *in wild-type and *nark *mutant plants**. (**a**, **b**) Relative transcript levels of *GmNF-YA1 *in shoots and root-parts of soybean plants 19 days (a) and 40 days (b) after partial inoculation with *R. irregularis *(for details see Figure 1 and legend of Figure 4). Transcript accumulation was determined by RT-qPCR and set in relation to *GmSUBI-1*. Data are presented as means + SD with *n *= 12-18 and *n *= 9-15 for wild-type and *nark *mutants, respectively, coming from three independent experiments. Different letters indicate significantly different values (*P *≤0.05, multiple Student's t-tests with Bonferroni correction). (**c**) Relative transcript levels of *GmNF-YA1 *in different tissues of non-inoculated 7-week-old wild-type, nts382, and nts1007 plants measured by RT-qPCR. Data are mean values + SD with *n *≥10 and *n *≥6 for wild-type and *nark *mutants, respectively, coming from two independent experiments. (**d**, **e**) Gene-specific transcript levels of *GmNF-YA1a *(d) and *GmNF-YA1b *(e) relative to *GmSUBI-1 *19 days after partial inoculation. RT-qPCR analyses were performed with gene-specific primers listed in Table S4 in the Additional file 1. Data are mean values + SD. Shoots: *n *≥3 of one experiment; root-parts: *n *≥9 of three independent experiments. Different letters indicate significantly different values (*P *≤0.05, multiple Student's t-tests with Bonferroni correction).

Next to the genes that seemed to be regulated by NARK AM-independently, we identified the targets of the Affymetrix probe set GmaAffx.40657.1.S1_at (named *GmNF-YA1*, see below) to be NARK-regulated in an AM-dependent manner (Figure 6). Transcript accumulation of these genes was significantly reduced by approximately 30% to 40% in autoregulated (non-colonized) roots of the mycorrhizal wild-type compared to roots of non-inoculated controls. Moreover, transcript levels were approximately 30% to 45% lower compared to non-colonized roots of both mycorrhizal *nark *mutants. This expression pattern was found 19 days (Figure 6a) as well as 40 days (Figure 6b) after inoculation of one root-part. In non-mycorrhizal plants, the highest mRNA levels of those genes were generally found in total root tissue and in root-hair zones (Figure 6c). Only relatively low mRNA levels were detected in above-ground tissues. There, no regulation by NARK or by AM was found (Figures 6a, b). Transcript levels were lowest in the youngest organs, namely in tips of roots and shoots, as well as in sink and source leaves (Figure 6c).

BLASTing the soybean genome [79] revealed that the target sequence of GmaAffx.40657.1.S1_at strongly aligns with transcript sequences of two homologous genes encoding putative CCAAT-binding TFs: Glyma03g36140 and Glyma19g38800. Both have an overall sequence identity of 90% to 94% and 86% to 91% on predicted coding sequence level and amino acid level, respectively (Figure S8 in Additional file 1). Ranges are due to alternative transcripts of Glyma19g38800. The putative amino acid sequences of both genes contain conserved protein domains supporting a function as CCAAT-binding TF of the NF-YA family (Figure S8b in Additional file 1). Thus, the homologous *TF *genes corresponding to GmaAffx.40657.1.S1_at were designated as *GmNF-YA1 *with *GmNF-YA1a *and *GmNF-YA1b *for Glyma03g36140 and Glyma19g38800, respectively. RT-qPCR analysis with gene-specific primers confirmed that both homologs, *GmNF-YA1a *and *GmNF-YA1b*, were AM-dependently suppressed by NARK in roots to a similar extent (Figure 6d, e).

The soybean transcription factor database v2.0 of the Center for Bioinformatics (CBI) at Peking University [80,81] contains in total 83 NF-Y proteins including 21 of the NF-YA family, 40 of the NF-YB family, and 22 TFs of the NF-YC family. According to the soybean Affymetrix GeneChip annotation of the NSF-funded project 'Gene Networks in Seed Development' [82], the array contains 36 probe sets targeting at least 34 different putative *NF-Y TF *genes (11x *NF-YA*, 10x *NF-YB*, 13x *NF-YC*). Our Affymetrix data did not indicate that one of the other putative *NF-Y *genes is NARK-regulated in autoregulated roots like *GmNF-YA1a/b *(Table S3 in Additional file 2). One putative *NF-YB *(Glyma20g00240, targeted by Gma.8502.1.S1_at) exhibited a higher mRNA level in non-mycorrhizal wild-type roots compared to mycorrhizal root-parts, however, root signals of non-mycorrhizal nts1007 were similar to that of the mycorrhizal wild-type contradicting a role in AOM (Figure S9a in Additional file 1). Another putative *NF-YB *(Glyma02g46970, targeted by Gma.12719.1.S1_at) showed slightly lower signals in shoots of mycorrhizal wild-type compared to shoots of non-mycorrhizal wild-type and of nts1007 (Figure S9b in Additional file 1). In contrast to the AM-dependently downregulated *GmNF-YA1a/b *genes, two putative *NF-Y *genes were found to be locally inducible by AM: Glyma09g07960 (Gma.3174.1.S1_at; NF-YA family) had slightly higher signals and Glyma12g34510 (GmaAffx.66953.1.S1_at; NF-YC family) was strongly induced in mycorrhizal root-parts (Figures S9c,d in Additional file 1). The closest homologs to *GmNF-YA1a/b*, Glyma02g35190 and Glyma10g10240 [41], appeared in our experimental setup not to be AM-induced, but developmentally regulated (Figure S10 in Additional file 1).

In addition to the here identified *NF-Y *genes regulated in roots of mycorrhizal plants, few *NF-Ys *were previously described to be locally induced by root endosymbionts. Most of them have been shown to be upregulated in nodulation, including the *NF-YA *gene*s *Glyma12g34510, Glyma02g35190, and Glyma10g10240 of soybean [49], *MtHAP2-1 *and *MtHAP2-2 *of *M. truncatula *[44-46,51], and *LjCBF-A01*/*LjNF-YA1 *and *LjCBF-A22 *of *L. japonicus *[43,52], the *NF-YB1 *gene from *L. japonicus *[52], as well as the *NF-YC *genes *PvNF-YC1 *and *PvNF-YC2 *of *Phaseolus vulgaris *[50,53] and MT007765 of *M. truncatula *[51]. AM-inducible *NF-YB *(Mtr.4282.1.S1_at) and *NF-YC *genes (*MtCBf1*/Mtr.51511.1.S1_at and *MtCbf2*/Mtr.16863.1.S1_at) were previously found in *M. truncatula *[48]. Promoter studies indicate a rather general role for these genes in the coordination of AM fungal colonization during all stages of AM [48]. However, AM-induced *NF-YA *genes or other *NF-Y *genes downregulated during mycorrhization have so far not been described.

### RNAi-mediated downregulation of *GmNF-YA1a/b *decreases AM

**Figure 7:**
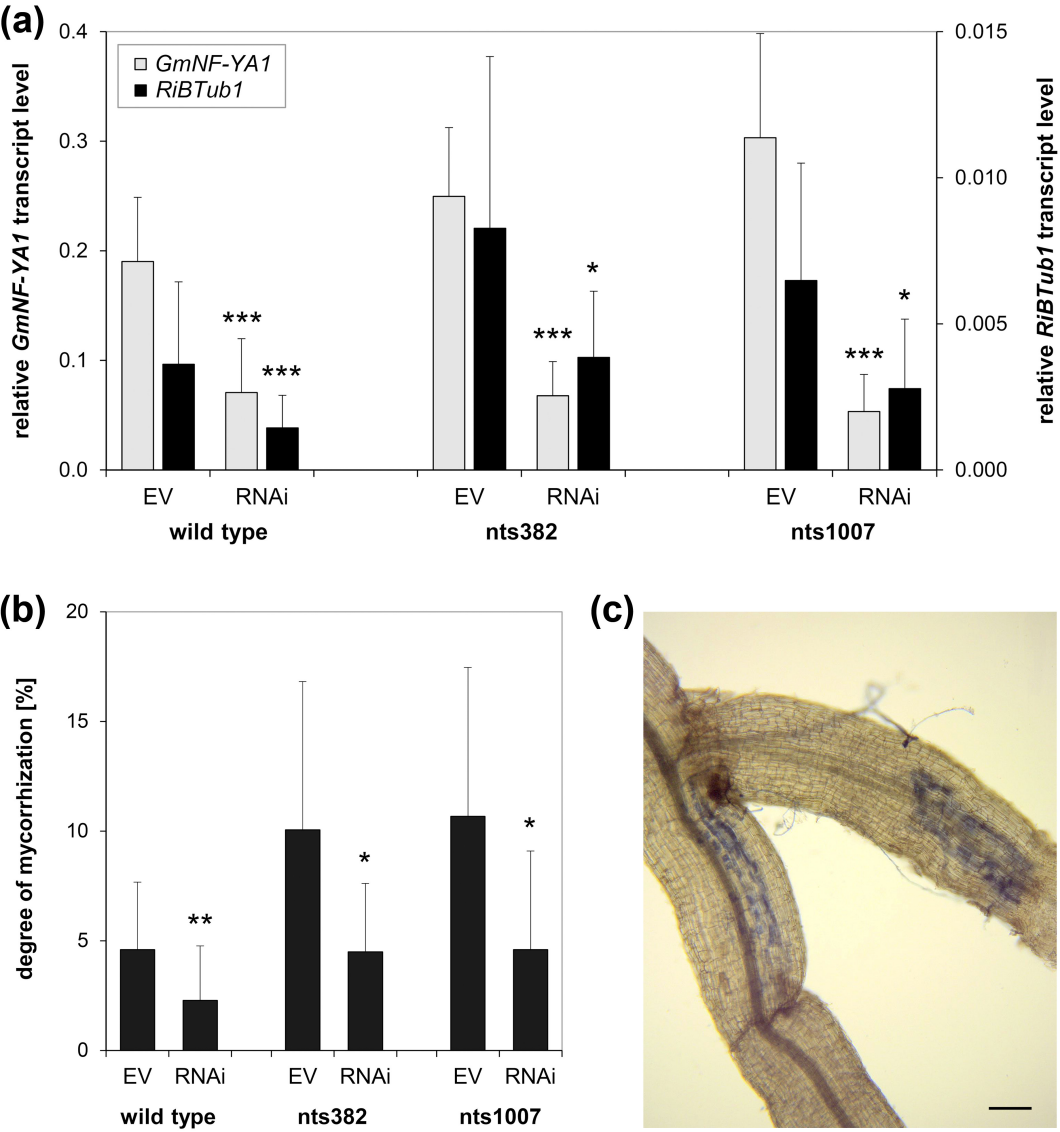
**Functional analysis of *GmNF-YA1 *in transgenic roots of chimeric soybean plants inoculated with *R***. *irregularis*. Gene expression of *GmNF-YA1a/b *was suppressed by an RNAi approach in *A. rhizogenes*-transformed roots of wild-type, nts382, and nts1007. Plants were harvested for analysis 3 weeks after AM fungal inoculation. (**a**, **b**) Analysis of the RNAi effect and the root colonization by *R. irregularis *in transformed roots. Transcript levels of *GmNF-YA1 *(gray columns) and *RiBTub1 *(black columns) were determined by RT-qPCR and are given in relation to *GmSUBI-1 *(a). For gene-specific transcript levels of *GmNF-YA1a *and *GmNF-YA1b *see Table 2. AM fungal colonization of roots was in addition analyzed microscopically after staining of roots (b). RNAi: roots transformed with *A. rhizogenes *carrying the RNAi construct. EV: empty vector control. Data are presented as means + SD with *n *≥30 and *n *≥15 for wild-type and *nark *mutants, respectively, coming from two independent experiments. Data of EV control and RNAi plants were pairwise compared by the Student's t-test. *P ≤0.05, **P ≤0.01, ***P ≤0.001. (**c**) AM fungal colonization caused by individual infection events. Bar represents 100 µm.

To test whether the NARK-suppressed *GmNF-YA1a/b *genes are involved in the process of mycorrhization, an RNAi construct was created to suppress *GmNF-YA1a/b *gene expression in roots of soybean. The *GmNF-YA1*-RNAi construct targets a 428 bp region of *GmNF-YA1a *and of *GmNF-YA1b *with 100% and 97% nucleotide sequence identity, respectively (Figure S8a in Additional file 1). Expression of *GmNF-YA1*-RNAi in roots of soybean wild-type, nts382, and nts1007 plants led to an reduced *GmNF-YA1 *mRNA level that was on average 63%, 73%, and 82%, respectively, lower than in the empty vector controls (Figure 7a). Both homologs were similarly suppressed (Table 2). Transcript analysis of the closest *GmNFYA-1 *homologs, Glyma10g10240 and Glyma02g35190 [41], revealed that the RNAi construct was almost specific to *GmNF-YA1a *and *GmNF-YA1b *(Table 2). Only Glyma02g35190 was significantly reduced by on average 35%. However, in wild-type roots, Glyma02g35190 as well as Glyma10g10240 were not found to be regulated during an AM interaction (Figure S10 in Additional file 1).

**Table 2:** Transcript analysis of *NF-YA *and *phosphate transporter *genes in *A.*

	EV			RNAi			Ratio
Gene	Mean		SD	Mean		SD	(RNAi/EV)
Glyma03g36140 (*GmNF-YA1a*)	0.21	±	0.097	0.084	±	0.048	0.40***
Glyma19g38800 (*GmNF-YA1b*)	0.19	±	0.099	0.082	±	0.055	0.44***
Glyma10g10240 (putative *NF-YA*)	0.021	±	0.014	0.017	±	0.015	0.80
Glyma02g35190 (putative *NF-YA*)	0.036	±	0.018	0.023	±	0.015	0.65**
Glyma13g08720 (*phosphate transporter*)	0.026	±	0.018	0.0098	±	0.0075	0.38***
Glyma14g28780 (*phosphate transporter*)	0.15	±	0.10	0.060	±	0.051	0.40***
Glyma14g36650 (*phosphate transporter*)	0.0026	±	0.0023	0.0010	±	0.0006	0.37***
*RiBTub1*	0.0036	±	0.0028	0.0014	±	0.0011	0.40***

*rhizogenes*-transformed roots

Transcript levels of different *NF-YA *genes and AM-induced *phosphate transporters *were exemplified analyzed in chimeric plants with a Bragg wild-type background. Transcripts relative to *GmSUBI-1 *were determined by RT-qPCR using gene-specific primers (for primer information see Table S4 in Additional file 1). Specificity of primer pairs was assessed by sequencing of RT-qPCR products. Data are mean values + SD with *n *= 30 and *n *= 35 for EV and RNAi, respectively, coming from two independent experiments. Data of empty vector control and RNAi plants were pairwise compared by the Student's t-test. *P ≤0.05, **P ≤0.01, ***P ≤0.001. Significant changes are highlighted in bold.

EV: empty vector control; RNAi: roots expressing the RNAi construct.

Simultaneously to reduced *GmNF-YA1a/b *mRNA accumulation, *GmNF-YA1*-RNAi expressing roots had on average a by 55% reduced colonization rate than the empty vector controls as determined by transcript analysis of the fungal marker gene *RiBTub1 *and by microscopic analysis. Such a reduced AM fungal colonization upon suppression of *GmNF-YA1 *mRNA accumulation occurred in both *nark *mutants as well as in wild-type plants (Figures 7a, b). Microscopy revealed that the mycorrhization was in a relatively early stage of establishment and that the rate of root colonization was mainly a consequence of individual infection events instead of excessive longitudinal spreading of the fungus after penetration into the root (Figure 7c). Thus, the degree of colonization indicates a lower number of infection events in RNAi plants than in the empty vector controls. To additionally assess the function of the AM symbiosis in the transformed roots, we measured transcript levels of AM-inducible phosphate transporters. In *M. truncatula*, *MtPT4 *represents a phosphate transporter gene that is specifically induced by AM and indispensable for the symbiosis [83,84]. Soybean phosphate transporters were previously analyzed identifying Glyma13g08720, Glyma14g28780, and Glyma14g36650 as closest homologs to *MtPT4 *[85] and as AM-inducible genes [86]. On average, the three AM-inducible phosphate transporter genes were suppressed by approximately 60% in roots expressing the *GmNF-YA1*-RNAi construct compared to the empty vector control (Table 2). These data clearly indicate a function of GmNF-YA1a/b as positive regulators in AM formation.

The decreased formation of AM in *GmNF-YA1*-RNAi roots is in line with the gene regulation of *GmNF-YA1a/b *observed in this study: expression of *GmNF-YA1 *was highest in non-inoculated roots (Figure 6c) and was systemically suppressed in wild-type roots during an AM interaction (Figure 6) thereby contributing to AOM leading to a reduced mycorrhizal colonization. Moreover, RNAi-mediated suppression of *GmNF-YA1a/b *in roots reduced AM formation not only in the wild-type but also in the *nark *mutant background pointing to the fact that GmNF-YA1a/b acts downstream of NARK.

## Conclusions

Overall, our gene expression analysis in split-root experiments provides for the first time insights into transcriptional regulation by NARK during the autoregulation of arbuscular mycorrhization - a systemic regulatory mechanism controlling the most widespread and ancient interaction between vascular terrestrial plants and microbes. By RT-qPCR analysis of plant material of independent experiments, we verified nine genes to be regulated by NARK. Gene expression of *annexins *indicate putative involvement in AM establishment and possibly also in regulation of AM. Furthermore, we identified *NF-YA *genes regulated during AM, including two NARK-regulated ones named *GmNYA1a *and *GmNYA1b*. So far, no information on a role of NF-YAs during AM symbiosis or autoregulation was available. By our transcript and functional gene analysis, we designate the newly identified TF subunits GmNF-YA1a/b as positive regulators of mycorrhization, which might act as targets for NARK-mediated AOM to restrict new infections events.

**Figure 8:**
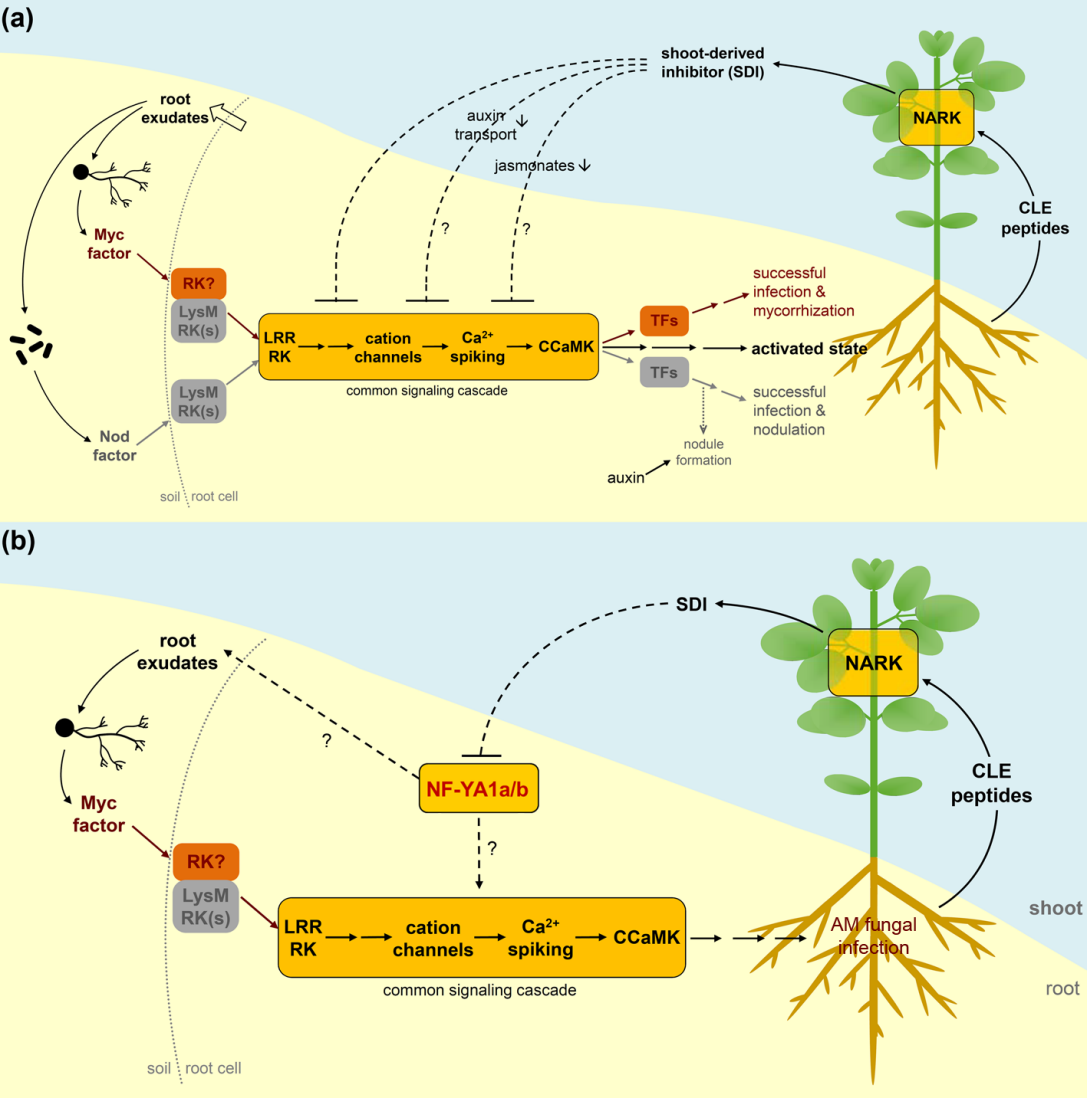
**Model of the autoregulation system in soybean and proposed function of GmNF-YA1a/b in AOM**. (**a**) Both, AM interaction and nodulation are based on an early signal exchange between the partners. Root exudates induce production of microbial signals (Myc/Nod factors) that are perceived by symbiosis-specific plasma membrane-bound receptor kinases (RKs). The signal is then translocated to the nucleus via activation of the common early signaling cascade finally leading to the induction of specific TFs that mediate successful mycorrhization or nodulation. Additionally, the so-called activated state of the root is induced resulting most likely in production of CLE peptides as putative root-derived signals activating NARK in the shoot. NARK reduces, probably via the shoot-derived inhibitor SDI, shoot-to-root auxin transport and JA biosynthesis in the shoot. Downstream of NARK unknown components finally suppress the common early signaling cascade leading to reduced subsequent infections with AM fungi and rhizobia. (**b**) Proposed model of GmNF-YA1a/b function in establishment and autoregulation in AM symbiosis. GmNF-YA1a/b promote formation of AM either via stimulating production of root exudates attracting AM fungi or by acting as positive regulator of one of the components of the early signal transduction cascade of AM establishment. After AM fungal infection, in soybean plants with functional NARK the gene expression of *GmNF-YA1a/b *is systemically downregulated in roots. This might be caused directly by SDI or by other signals downstream of SDI.

How GmNF-YA1a/b promote AM, however, is still unclear. A stimulation of AM formation by GmNF-YA1a/b might either be done by promoting gene expression of one of the elements of the (common) early signaling cascade or by stimulating the production of root exudates that attract AM fungi (Figure 8). Previous studies on *M. sativa *split-root plants showed that inoculation with the AM fungus *Funneliformis mosseae *suppresses the production of some isoflavonoids including ononin in autoregulated root-parts [87]. In turn, application of ononin to autoregulated root-parts enhanced the colonization of such roots with *F. mosseae *[87]. It is tempting to speculate that GmNF-YA1a/b activates transcription of isoflavonoid biosynthesis genes. Another possible function comprises the interaction of GmNF-YA1a/b with Myc factor- or AM-induced NF-Y subunit(s) to drive gene expression that is required for successful AM fungal infection. Next to the induction of gene expression, however, NF-Y TFs can also repress transcription. Thus, GmNF-YA1a/b might potentially suppress gene expression that in return inhibit AM establishment. However, details of GmNF-YA1a/b function in establishment and regulation of mycorrhizal symbiosis and a possible function of GmNF-YA1a/b in nodulation will be the focus of future work. Our findings presented here indicate a new function of NF-YAs in the regulation of plant-microbe interactions, probably by supporting the early signal exchange between both partners.

## Materials and methods

### Plant material, germination of seeds, and growth conditions

Seeds of soybean cv. Bragg (wild-type and two allelic *nark *mutant lines, nts382 and nts1007) were surface-sterilized with 1.5% sodium hypochlorite for 5 min. After washing with distilled water, seeds were germinated in wet expanded clay of 2 mm to 5 mm particle size (Original LamstedtTon; Fibo ExClay, Lamstedt, Germany). The same substrate was used for inoculum production and all experiments described below. If not mentioned otherwise, plants were grown in a plant growth chamber under 16 h light (200 µmol m^-2 ^s^-1^) at 26°C and 8 h dark at 22°C with a relative humidity of 60% to 65%. All plants were watered with distilled water and fertilized with Long Ashton nutrient solution containing 20% of the regular phosphate content [88].

### Fungal material and inoculation of plants

The AM fungus *R. irregularis *(formerly *Glomus intraradices *[89]) Schenk & Smith isolate 49 [90] was enriched by previous co-cultivation with leek (*Allium porrum *cv. Elefant) in a greenhouse. As inoculum, the freshly harvested substrate was used. Roots or root-parts of soybean plants were inoculated with *R. irregularis *by careful removal of the previous substrate and transfer into expanded clay containing 10% to 20% (v/v) *R. irregularis*-inoculum. Non-mycorrhizal roots or root-parts were transferred in the same way into pure expanded clay.

### Split-root experiments

In total, three independent split-root experiments (I-III) were performed. In each experiment, Bragg wild-type, nts382, and nts1007 plants were cultivated in parallel as follows: after 6 days of germination, the main root of the seedlings was cut off around 2 cm below the shoot basis, and seedlings were continued to be cultivated under the conditions described. After 2 days, plants were transferred to the split-root system by dividing the arising lateral roots onto two individual pots connected to each other (each 9 × 9 × 9.5 cm) (Figure S1 in Additional file 1). In doing so, mycorrhizal plants were inoculated on one root-part with 20% (v/v) inoculum. The other root-part and root-parts of control plants were not inoculated (Figure 1). Nineteen days after inoculation, 50% of the plants were harvested.

To check plants for AOM activation at the first harvest time-point, the non-harvested plants were transferred to bigger pots (each 11 × 11 × 11.5 cm). In doing so, the previously non-inoculated root-part of mycorrhizal plants and one root-part of control plants were inoculated with 10% (v/v) inoculum (Figure 1 and Figure S1 in Additional file 1). Plants were harvested 21 days after subsequent inoculation.

In experiment II and III some additional control and mycorrhizal plants were cultivated in bigger pots without subsequent inoculation and harvested 40 days after inoculation, and above- and below-ground plant tissues were harvested as described below.

### Root transformation experiments

For RNAi suppression of *GmNF-YA1a/b*, a fragment of *GmNF-YA1 *of 428 bp size was amplified using PCR Super Mix High Fidelity (Invitrogen, Carlsbad, CA, USA) (for primer binding sites see Figure S8a in Additional file 1). To allow directional cloning of the fragment in sense and antisense direction, the restriction sites *Bam*HI, *Spe*I and *Swa*I, *Asc*I, respectively, were attached during PCR (for primer sequences see Table S4 in Additional file 1). Gel-purified fragments were ligated into the pGEM-T Easy vector (Promega, Madison, WI, USA). Complementary sense and antisense arms, showing 100% nucleotide identity to Glyma03g36140, were cloned into the pRNAi vector [91] under the CaMV 35S promoter. The resulting construct was cloned into the pRedRootII vector that contains the gene for DsRed1 in the T-DNA region (kindly provided by R. Geurts, Wageningen, The Netherlands) using *Kpn*I and *Pac*I, and finally transformed into *A. rhizogenes *K599 [92]. As control, *A. rhizogenes *K599 cells were transformed with the empty pRedRootII vector.

Root transformation was performed according to the protocol published by Kereszt *et al. *[92] using a bacterial suspension that was injected into the hypocotyl close to the cotyledonary node of 5-day-old-seedlings of soybean wild-type, nts382, and nts1007. All seedlings were kept in a humid chamber under 100% humidity at 12 h light (200 µmol m^-2 ^s^-1^, 28°C) and 12 h dark (25°C). After 3 and 4 weeks, newly developed hairy roots were screened twice for fluorescence of DsRed1 using a fluorescence stereomicroscope equipped with a DsRed filter (Leica 409 MZ FLIII; Leica Microsystems, Wetzlar, Germany). Two to four hairy roots that showed the strongest fluorescence were kept, and the other hairy or wild-type roots were removed. Hairy roots were covered with expanded clay and plants were further cultivated in a greenhouse (16 h sun light supplemented with artificial light, 22°C; 8 h dark, 20°C) acclimating them slowly to lower humidity. One day after second screening, all plants were inoculated with 15% (v/v) inoculum, further cultivated in the greenhouse, and harvested 3 weeks later.

### Harvest of plants and plant tissues, isolation of total RNA

Split-root plants were rapidly taken out of the substrate, which was carefully removed from the root-parts. Root-parts and shoots were separated and immediately flash frozen in liquid nitrogen. Samples were homogenized in liquid nitrogen and stored at -80°C until extraction. Roots of *A. rhizogenes*-transformed plants were harvested in the same way. For staining of fungal structures (see below), a median cross-section of each root sample of approximately 1 cm length was taken before freezing.

Different tissues of some non-inoculated plants of split-root experiment II and III were harvested 7 weeks after sowing. For that purpose, shoots were rapidly dissected into shoot tips, young (sink) leaves, fully developed (source) leaves, yellowing (senescing) leaves, and stalks. For each plant, roots were rapidly removed from the expanded clay and six to ten root-tips, six to ten root-hair zones, and the remaining root material were selected. All tissues were immediately flash frozen, homogenized in liquid nitrogen and stored at -80°C.

Total RNA was isolated using the Qiagen RNeasy Plant Mini Kit (Qiagen, Hilden, Germany) including DNA digestion (RNase-free DNase Set; Qiagen) according to the supplier's protocol.

### Staining of fungal structures and analyzing the degree of mycorrhization

Colonization with *R. irregularis *was analyzed in a representative cross-section of each root sample. Fungal structures in the root pieces of around 1 cm length were stained according to Vierheilig *et al. *[93] using 5% (v/v) ink (Sheaffer Skrip jet black; Sheaffer Manufacturing, Shelton, CT, USA) in 2% (v/v) acetic acid. The degree of mycorrhization was determined for at least 50 root-pieces per root sample using a stereomicroscope (Stemi 2000-C; Carl Zeiss, Jena, Germany). In root-transformation experiments, all root-pieces per root sample were analyzed. Pictures were taken with a digital microscope system (VHX-1000 equipped with a VH-Z250R zoom lens, Keyence, Osaka, Japan).

### Affymetrix analysis

RNA samples of three plants per treatment of the split-root experiment I were analyzed using Affymetrix GeneChips. Synthesis and purification of cDNA; synthesis, labeling, purification, quality control, and fragmentation of cRNA; and hybridization, washing, and scanning of the chips were done by the Affymetrix service partner Atlas Biolabs (Berlin, Germany) according to the supplier's protocols. Data of the binary files were processed and analyzed using the DNA-chip analyzer software package dChip [57]. Arrays were normalized on perfect match and mismatch probe level separately for roots and shoots with a root/shoot array with median overall intensity as baseline array. Afterwards, the model-based expression values were calculated for all arrays together. To find genes that are regulated by AM and NARK, pairwise comparisons and combined pairwise comparisons were performed with dChip. If not mentioned otherwise, the recommended presettings of the software were used. At first, we searched for *R. irregularis-*regulated genes in wild-type and in the nts1007 mutant. To find genes locally induced or suppressed after *R. irregularis*-inoculation, we performed the following comparisons, independently for wild-type and nts1007: mycorrhizal versus non-mycorrhizal root-parts of mycorrhizal plants and *versus *non-mycorrhizal roots of control plants (Figure 3a). To find genes systemically induced or suppressed after *R. irregularis*-inoculation, comparisons were performed as follows, again each for wild-type and nts1007: (I) shoots of mycorrhizal plants *versus *shoots of non-mycorrhizal plants (Figure 3b), (II) non-inoculated (autoregulated) root-parts of mycorrhizal plants *versus *roots of non-inoculated control plants (Figure 3c). The criteria for all comparisons performed with dChip were larger than two-fold changes with *P *≤0.1 (unpaired and paired t-tests). For nts1007, only those genes were counted, which showed changes compared to control nts1007 as well as to control wild-type tissue. To avoid under-estimation of the standard error of group means, the measurement error was considered leading to less significant *P *values than the standard analysis. The identification of additional candidate genes was performed using the clustering software CLANS [65].

Putative gene functions and categorizations are in accordance with the soybean Affymetrix GeneChip annotation of the NSF-funded project 'Gene Networks in Seed Development' [82]. Candidate genes without annotation were analyzed by sequence homology via searching the NCBI nucleotide collection with the BLAST tool [94]. Selected candidate genes (Table S1 in Additional file 2) were further analyzed by RT-qPCR. Target genes of Affymetrix probe sets were checked by BLAST analysis of the Affymetrix target sequence against the soybean genome [79] and updated with the latest version (Phytozome v9.0) [95].

The entire Affymetrix GeneChip data from this study are publicly available at the plant expression database PLEXdb [96] under the accession number GM53 [97] and at the Gene Expression Omnibus repository [98] under the GEO Series accession number GSE44685 [99].

### Determination of *R. irregularis *and soybean transcripts using RT-qPCR

Transcript levels of putative NARK-regulated soybean genes and of *R. irregularis β-Tubulin1 *(*RiBTub1*, GenBank:AY326320.1; for primer information see Table S4 in Additional file 1) were quantified by RT-qPCR. First strand cDNA synthesis of 1 µg RNA was performed in a final volume of 20 µL with M-MLV Reverse Transcriptase, RNase H Minus, Point Mutant (Promega) according to the supplier's protocol using oligo(dT)19 primer. RT^- ^samples were prepared in the same way using water instead of enzyme. QPCR primers for soybean candidate genes were designed with the Primer Express software (Applied Biosystems, Foster City, CA, USA) using the corresponding target sequences of the soybean Affymetrix GeneChip (for primer sequences see Table S1 in Additional file 2; primers of validated NARK-regulated genes are also listed in Table S4 in Additional file 1). As reference, soybean ubiquitin *GmSUBI-1 *transcripts were measured [GenBank:NM_001248971.1] (for primer information see Table S4 in Additional file 1).

For qPCR, 3 μL of 1:10 diluted cDNA were mixed with Brilliant II SYBR Green QPCR Master Mix (Agilent Technologies, Santa Clara, CA, USA), supplemented with the reference dye ROX (final concentration: 30 nM), 2 pmol of forward primer and 2 pmol of reverse primer in a final volume of 10 μL in two to three independent technical replicates. As negative controls, water and RT^- ^samples (1:10 diluted) were used instead of cDNA. Fluorescence of SYBR Green I and of ROX were measured using the Mx3000P and Mx3005P QPCR systems (Agilent Technologies) and the following PCR program: enzyme activation (95°C for 10 min), 40 cycles of amplification (95°C for 30 s, 60°C for 1 min with measurement of fluorescence at the end of this step), followed by dissociation (95°C for 1 min, 60°C for 30 s, heating up to 95°C with a heating rate of 0.1°C s^-1 ^and continuous measurement). Data were evaluated with the MxPro software (Agilent Technologies). For each sample, mRNA levels of target genes were normalized to *GmSUBI-1 *mRNA by the comparative Ct (2^-ΔCt^) method [100].

### Statistical analysis

All data are derived from at least three biological replicates per experiment. The exact number of replicates used for each analysis is given in the corresponding figure legend. If not mentioned otherwise, data are tested for significant differences with *P *≤0.05 by the Student's t-tests. In case of multiple testing, Bonferroni correction was applied.

### Data access

Supplemental material is available for this article. The Affymetrix GeneChip data from this study have been submitted to the plant expression database PLEXdb [96] and are available under the accession number GM53 [97] and have in addition been deposited in the Gene Expression Omnibus [98] accessible through GEO Series accession number GSE44685 [99].

## Abbreviations

AM: arbuscular mycorrhiza; AOM: autoregulation of mycorrhization; NF-Y: nuclear factor Y; RK: receptor kinase; RNAi: RNA interference; RT-qPCR: reverse transcriptase quantitative polymerase chain reaction; TF: transcription factor.

## Competing interests

The authors declare that they have no competing interests.

## Authors' contributions

SS, PMG, and BH designed the research and discussed the data. SS performed the research, analyzed the data, and wrote the manuscript. All authors have read and approved the manuscript for publication.

## Supplementary Material

Additional file 1 **Supplemental Figures S1 to S10 and Supplemental Table S4: Figure S1**. Procedure and plant photographs of split-root experiments. Figure S2. Mycorrhization phenotype of wild-type, nts382, and nts1007 in the split-root experiments II and III. Figure S3. Mycorrhization phenotype of wild-type, nts382, and nts1007 in dependence on phosphate and nitrate fertilization. Figure S4. Putative NARK-response genes identified by Affymetrix GeneChip analysis. Figure S5. Additional putative NARK-response genes in soybean plants 19 days after inoculation. Figure S6. Putative NARK-response genes in 7-week-old soybean plants. Figure S7. Affymetrix gene expression data of putative *annexins *19 days after inoculation. Figure S8. Sequence information of the putative *CCAAT-binding TF *genes targeted by GmaAffx.40657.1.S1_at. Figure S9. Affymetrix gene expression data of other putative *NF-Y *genes in soybean plants 19 days after inoculation. Figure S10. Transcripts accumulation of the putative *NF-YA *genes Glyma10g10240 and Glyma02g35190 in root tissue upon *R. irregularis*-inoculation. Table S4. Sequences of primers used for RT-qPCR analysis and for creating the RNAi construct. Click here for file

Additional file 2 **Supplemental Tables S1 to S3: **Table S1. Soybean genes found in the Affymetrix data to be locally regulated by *R. irregularis *19 days after inoculation. Table S2. Soybean candidate genes selected by Affymetrix data for further analyses. Table S3. Affymetrix data of putative *CCAAT binding TF *genes of soybean. Click here for file
