# Supplementary material for: Analyzing the soybean transcriptome during autoregulation of mycorrhization identifies the transcription factors GmNF-YA1a/b as positive regulators of arbuscular mycorrhization
Source: Genome Biol. 2013 Jun 18;14(6):R62. doi: 10.1186/gb-2013-14-6-r62 (PMC3706930; doi:10.1186/gb-2013-14-6-r62)
Supplement: Additional file 1 — Supplemental Figures S1 to S10 and Supplemental Table S4: Figure S1. Procedure and plant photographs of split-root experiments. Figure S2. Mycorrhization phenotype of wild-type, nts382, and nts1007 in the split-root experiments II and III. Figure S3. Mycorrhization phenotype of wild-type, nts382, and nts1007 in dependence on phosphate and nitrate fertilization. Figure S4. Putative NARK-response genes identified by Affymetrix GeneChip analysis. Figure S5. Additional putative NARK-response genes in soybean plants 19 days after inoculation. Figure S6. Putative NARK-response genes in 7-week-old soybean plants. Figure S7. Affymetrix gene expression data of putative annexins 19 days after inoculation. Figure S8. Sequence information of the putative CCAAT-binding TF genes targeted by GmaAffx.40657.1.S1_at. Figure S9. Affymetrix gene expression data of other putative NF-Y genes in soybean plants 19 days after inoculation. Figure S10. Transcripts accumulation of the putative NF-YA genes Glyma10g10240 and Glyma02g35190 in root tissue upon R. irregularis-inoculation. Table S4. Sequences of primers used for RT-qPCR analysis and for creating the RNAi construct. [file gb-2013-14-6-r62-S1.PDF]

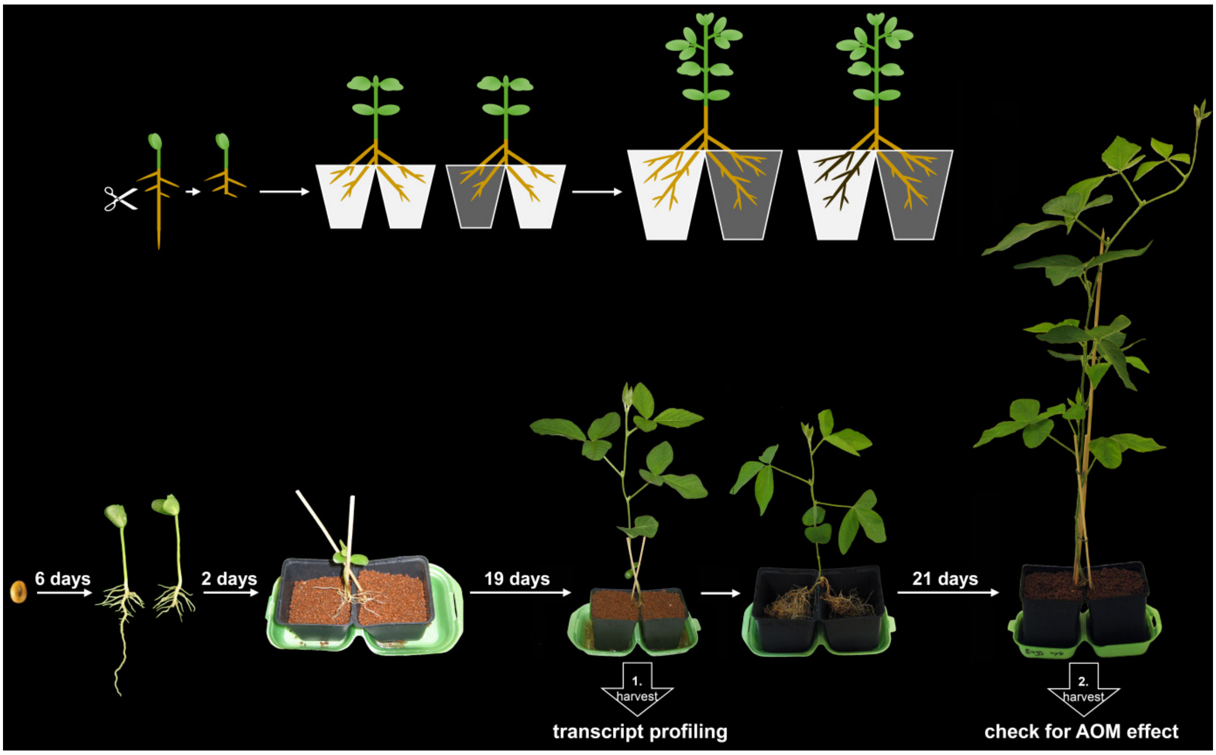

Figure S1. Procedure and plant photographs of split-root experiments.

Plants of soybean wild type, nts382 and nts1007 were set-up as split-root plants as described in the Materials and methods section and in Figure 1. The photographs show representative plants at time-point of cutting off the main root (6 days after germination of seeds), of transferring the plants into the split-root system (after 2 days of recovery), of first harvest or subsequent inoculation (19 days after initial inoculation with *R. irregularis* at the first root-part) and of second harvest (21 days after subsequent inoculation of second root-parts). The schematic drawing illustrates the inoculation of different root-parts with *R. irregularis* (indicated by the dark color, see also Figure 1).

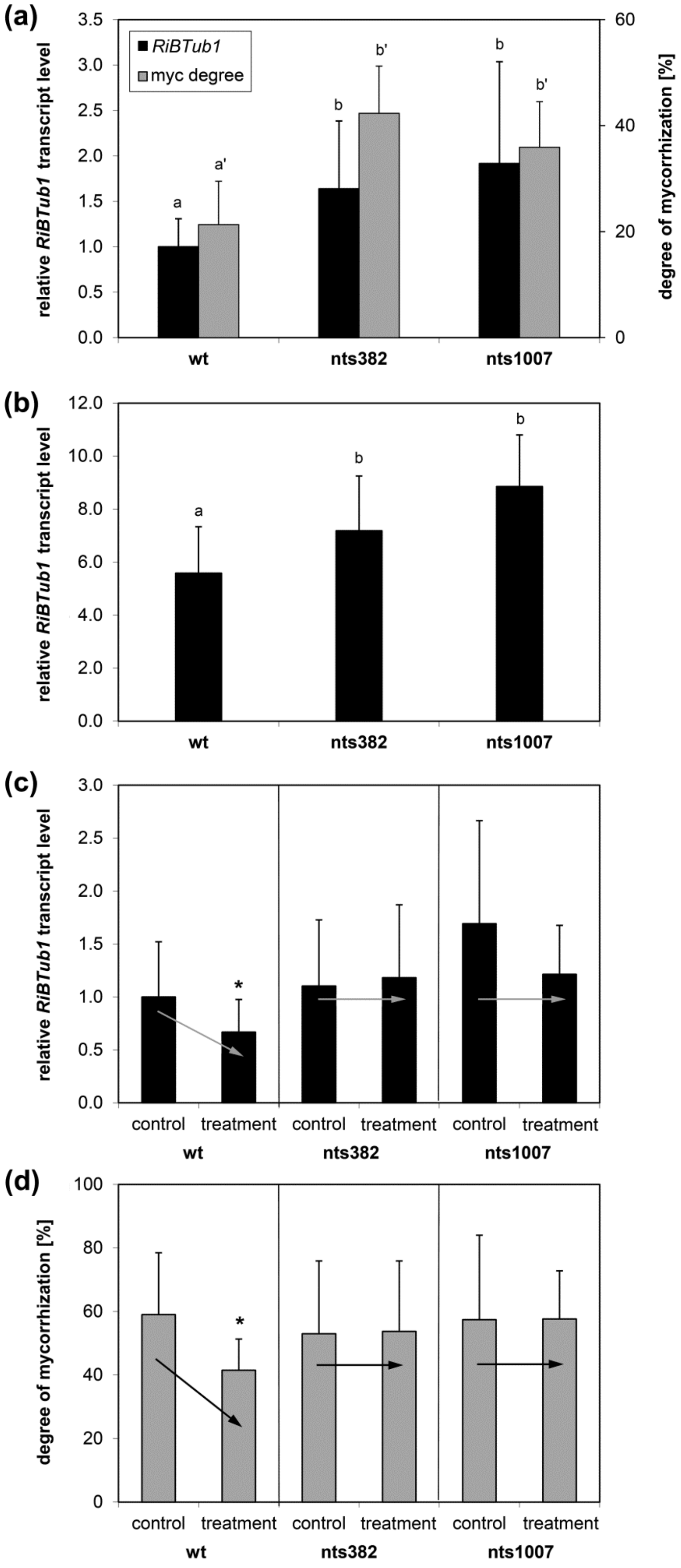

Figure S2. Mycorrhization phenotype of wild type, nts382, and nts1007 in the split-root experiments II and III.

(a, b) Colonization of initially inoculated root-parts at time-point of first harvest (= 19 days after inoculation with *R. irregularis*) (a) and at time-point of second harvest (= 40 days after inoculation with *R. irregularis*) (b).

(c, d) Colonization of subsequently inoculated root-parts at time-point of second harvest (= 21 days after inoculation with *R. irregularis*).

The colonization of root-parts by *R. irregularis* was analyzed microscopically after staining of roots (degree of mycorrhization) (a, d) and/or by transcript analysis of the *R. irregularis* marker gene *RiBTub1* (a-c). Transcript levels of *RiBTub1* were normalized to transcript levels of the soybean ubiquitin gene *GmSUBI-1*. Afterwards, for each split-root experiment, relative *RiBTub1* transcript levels were set in relation to the mean values of initially inoculated root-parts of treated wild-type plants (a) or of subsequently inoculated root-parts of wild-type control plants (b, c). All data are given as mean values of ≥12 plants of two independent experiments + SD. Data of initially inoculated root-parts (a, b) were statistically analyzed by multiple Student's t-tests with Bonferroni correction; different letters designate significant differences with $P \leq 0.05$. Data of subsequently inoculated root-parts (c, d) were pairwise compared between control and mycorrhizal plants ('treatment'), each for wild type, nts382 and nts1007, by the Student's t-test. *$P \leq 0.05$.

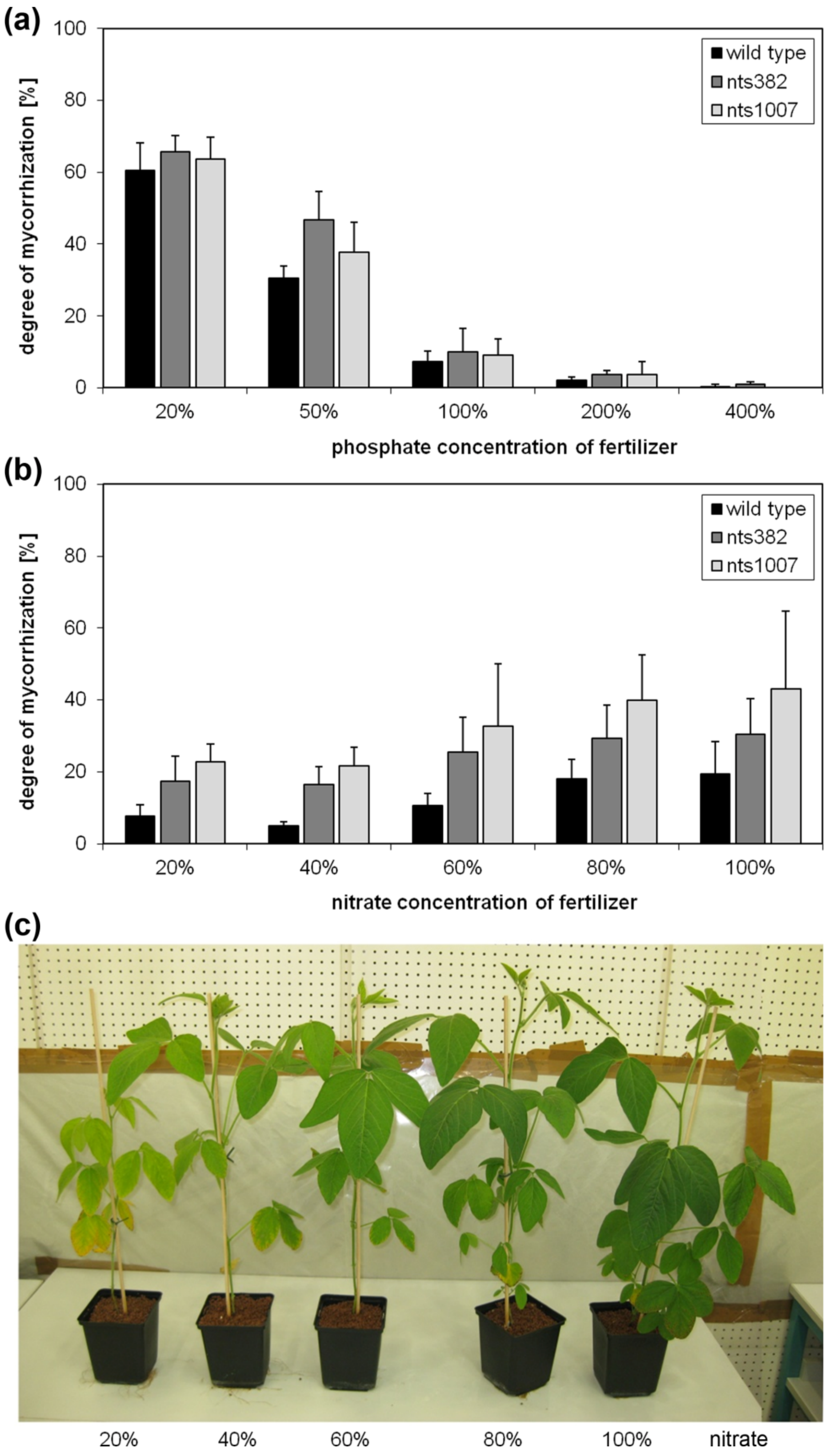

Figure S3. Mycorrhization phenotype of wild type, nts382, and nts1007 in dependence on phosphate and nitrate fertilization.

(a, **b)** Degree of mycorrhization of plants fertilized with different amounts of phosphate (a) or nitrate (b). Plants of both experiments were grown up and cultivated as described for the split-root experiments but without dividing the roots on two compartments. Instead, 10-day-

old plants were transferred each to one pot (9 x 9 x 9.5 cm) and inoculated with 20% (v/v) *R. irregularis* inoculum. Twice per week, plants were fertilized with 25 ml 10x Long Ashton nutrient solution [88], supplemented with different amounts of phosphate or nitrate given in relation to the regular amounts of phosphate and nitrate (=100%). For testing distinct phosphate concentrations, 20%, 50%, 100%, 200%, and 400% of the regular phosphate content was used. To avoid putative nitrate limitation at 400% phosphate concentration (which might in return stimulate mycorrhization), 200% of nitrate was used; all other plants of the phosphate test were fertilized with 100% regular nitrate concentration. To induce nitrate deficiency, concentrations of 20%, 40%, 60%, 80%, and 100% of nitrate were used; phosphate concentration was constant at 100% to avoid additional phosphate depletion. Plants were harvested 4 weeks after inoculation. The degree of mycorrhization was analyzed in a representative cross-section of each root system, in which fungal structures were stained as described in the Materials and methods section. Data are presented as mean values + SD (n = 5).

(c) Picture of representative plants fertilized with different nitrate concentrations taken prior to harvest.

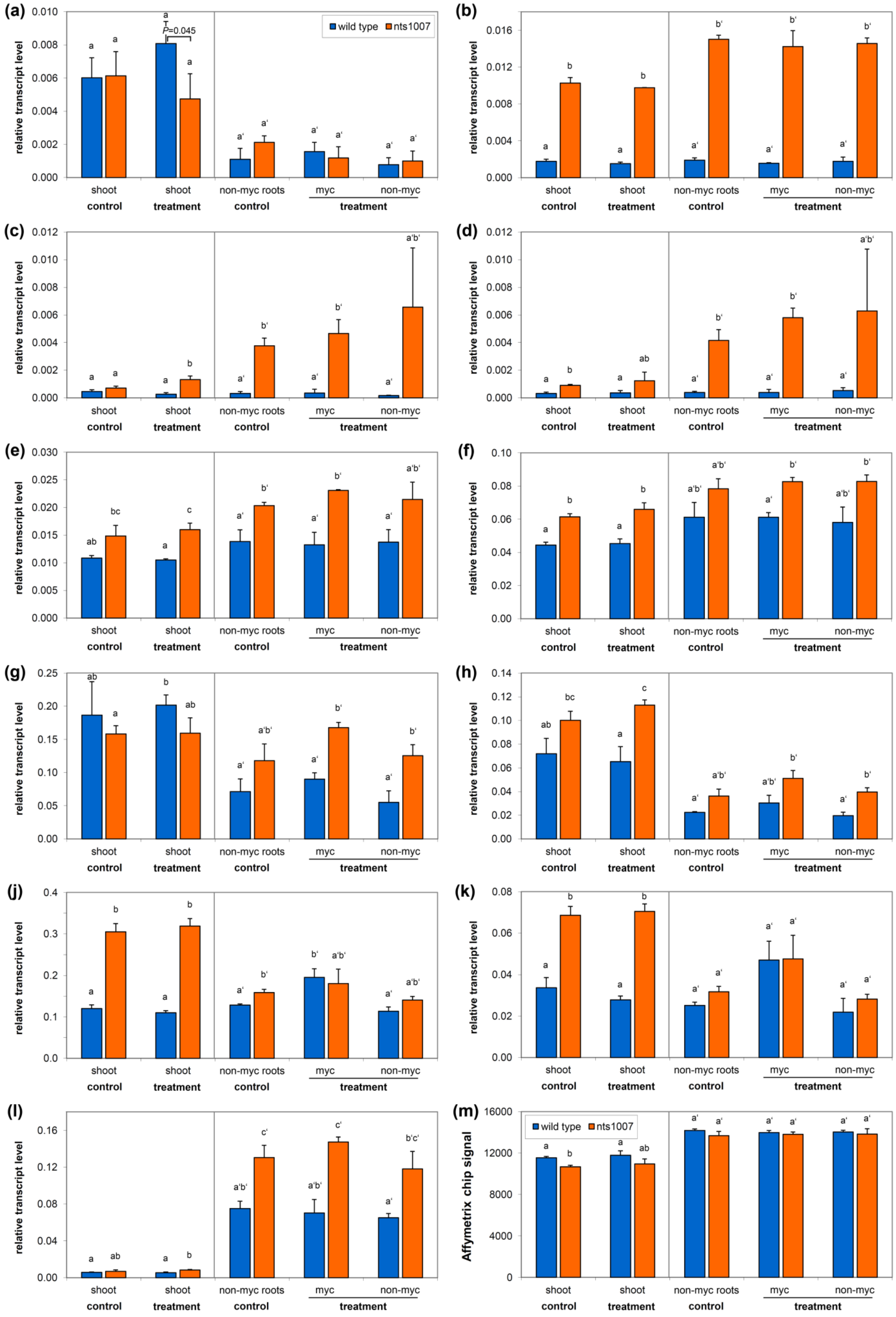

Figure S4. Putative NARK-response genes identified by Affymetrix GeneChip analysis.
(a) GmaAffx.30002.1.S1_at targeting Glyma15g15171 (unknown).

(b) Gma.7686.1.S1_at targeting Glyma18g17440 (putative *ornithine acetyl transferase*).
(c) GmaAffx.82595.1.S1_at targeting Glyma02g11150 (*GmSIK1*).
(d) GmaAffx.82595.2.S1_at targeting Glyma02g11150 (*GmSIK1*).
(e) GmaAffx.68580.1.S1_at targeting Glyma17g09270 (putative *DEAD box RNA helicase*).
(f) GmaAffx.46141.1.S1_at targeting Glyma17g09270 and Glyma05g02590 (putative *DEAD box RNA helicases*).
(g) Gma.6487.1.A1_at targeting Glyma10g35000 (unknown).
(h) Gma.17992.1.S1_at targeting Glyma07g36986 (unknown).
(j) Gma.3440.2.S1_at targeting Glyma15g38010 (putative *annexin*).
(k) Gma.3440.2.S1_a_at targeting Glyma13g26960 and Glyma15g38010 (putative *annexins*).
(l) GmaAffx.40657.1.S1_at targeting Glyma03g36140 and Glyma19g38800 (putative *NF-YAs*).
(m) GmaAffx.93644.1.S1_x_at targeting Glyma20g27950 (*GmSUBI-1*).
Transcript accumulation was determined by Affymetrix GeneChip analysis in wild-type and *nark* mutant nts1007 plants harvested 19 days after initial inoculation with *R. irregularis*. Mycorrhizal plants ('treatment') were inoculated only on one root-part (myc) as shown in Figure 1. The other root-part stayed non-inoculated (non-myc). Control plants were completely non-mycorrhizal. Relative transcript levels were calculated by setting Affymetrix chip signals for putative NARK-response genes (a-k) in relation to chip signals for *GmSUBI-1* (l). For Affymetrix chip signals of putative NARK-regulated genes see Table S2 in Additional file 2. Data are mean values + SD (n = 3). Different letters indicate significant differences ($P \leq 0.05$, multiple Student's t-tests with Bonferroni correction performed separately for shoots and root-parts). The given P-value in (a) derived from a pair-wise comparison.

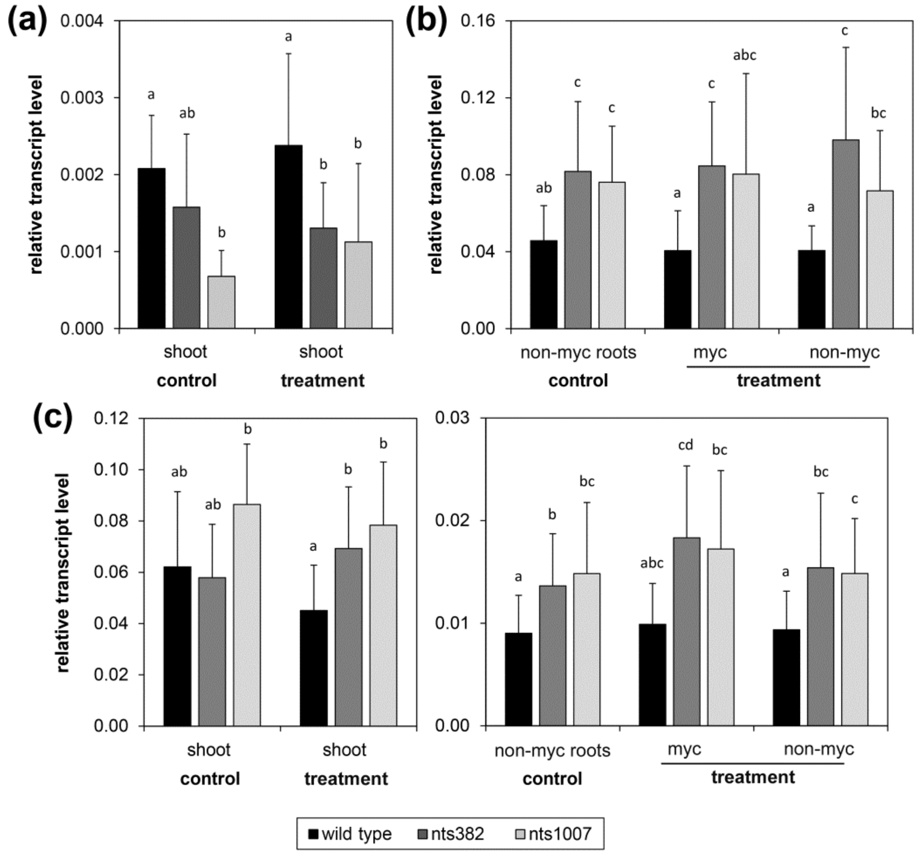

Figure S5. Additional putative NARK-response genes in soybean plants 19 days after inoculation.

(a) Glyma15g15171, unknown function.

(b) Glyma10g35000, unknown function.

(c) Glyma07g36986, unknown function.

Transcript levels relative to *GmSUBI-1* were determined by RT-qPCR in wild-type and *nark* mutant (nts382 and nts1007) plants harvested 19 days after initial inoculation with *R. irregularis* (for details see Figure 1). Data are mean values + SD with n = 9-18 and n = 8-15 for wild type and *nark* mutants, respectively, coming from at least two independent experiments. Different letters indicate significant differences ($P \leq 0.05$, multiple Student's t-tests with Bonferroni correction).

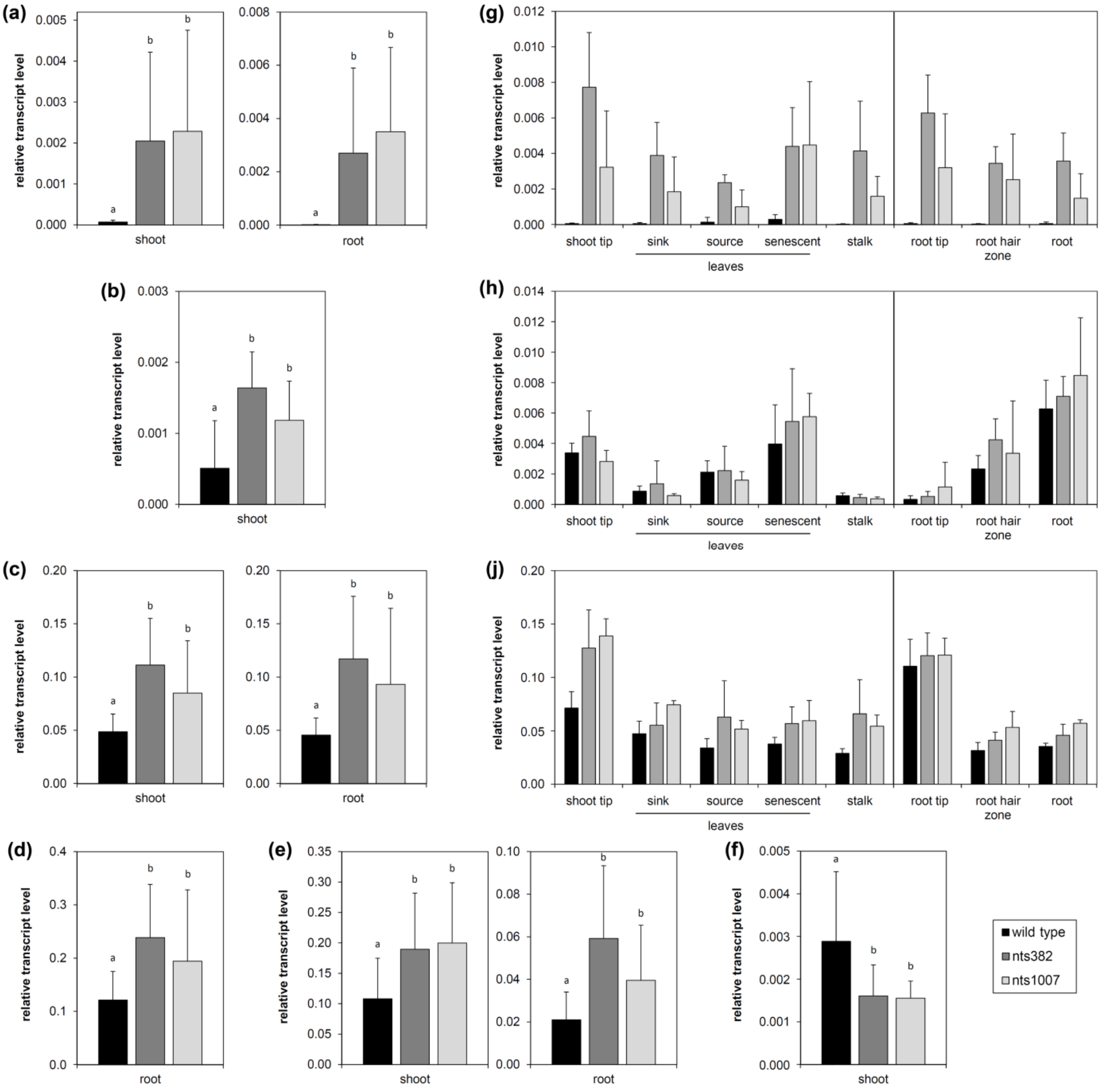

Figure S6. Putative NARK-response genes in 7-week-old soybean plants.

(a-f) Relative transcript levels in shoots and/or roots of mycorrhizal wild-type, nts382 and nts1007 plants 40 days after inoculation. Transcript levels relative to *GmSUBI-1* were determined by RT-qPCR. Data are means + SD with $n \geq 10$ and $n \geq 8$ for wild type and *nark* mutants, respectively, coming from two independent experiments. Different letters indicate significant differences ($P \leq 0.05$, multiple Student's t-tests with Bonferroni correction).

(g-j) Relative transcript levels in different tissues of non-inoculated 7-week-old wild-type and *nark* mutant (nts382 and nts1007) plants. (g) Mean values + SD with n = 10 and n = 6-7 for wild type and *nark* mutants, respectively, coming from two independent experiments. (h, j) Mean values + SD of plants of one experiment; wild type: n = 5-6, *nark* mutants: n = 3.

(a, **g)** Glyma18g17440, putative *ornithine acetyl transferase*.

(b, **h)** Glyma02g11150, *Stress-induced receptor-like kinase* (*GmSIK1*).

(c, **j)** Glyma17g09270, putative *DEAD box RNA helicase*.

(d) Glyma10g35000, unknown function.

(e) Glyma07g36986, unknown function.

(f) Glyma15g15171, unknown function.

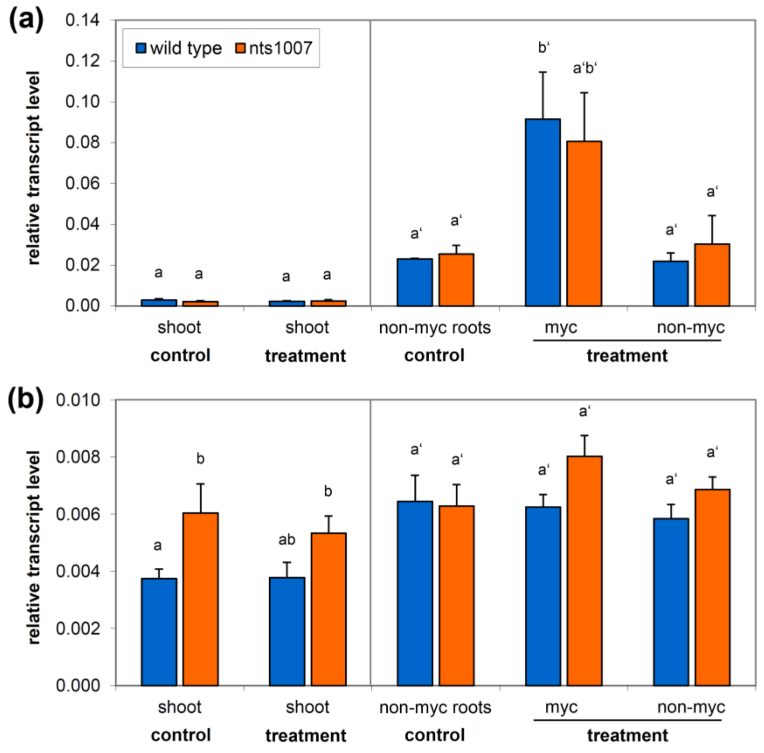

Figure S7. Affymetrix gene expression data of putative *annexins* 19 days after inoculation.

(a) GmaAffx.1082.1.S1_at targeting the predicted *annexin* Glyma04g27100.

(b) GmaAffx.1082.1.A1_at targeting the predicted *annexin* Glyma11g21457.

Affymetrix chip signals for putative *annexin* genes were set in relation to chip signals for GmaAffx.93644.1.S1_x_at targeting *GmSUBI-1* (see Figure S4m). Data are means + SD of three biological replicates. For details see the Materials and methods section. Different letters indicate significant differences ($P \leq 0.05$, multiple Student's t-tests with Bonferroni correction performed separately for shoots and root-parts).

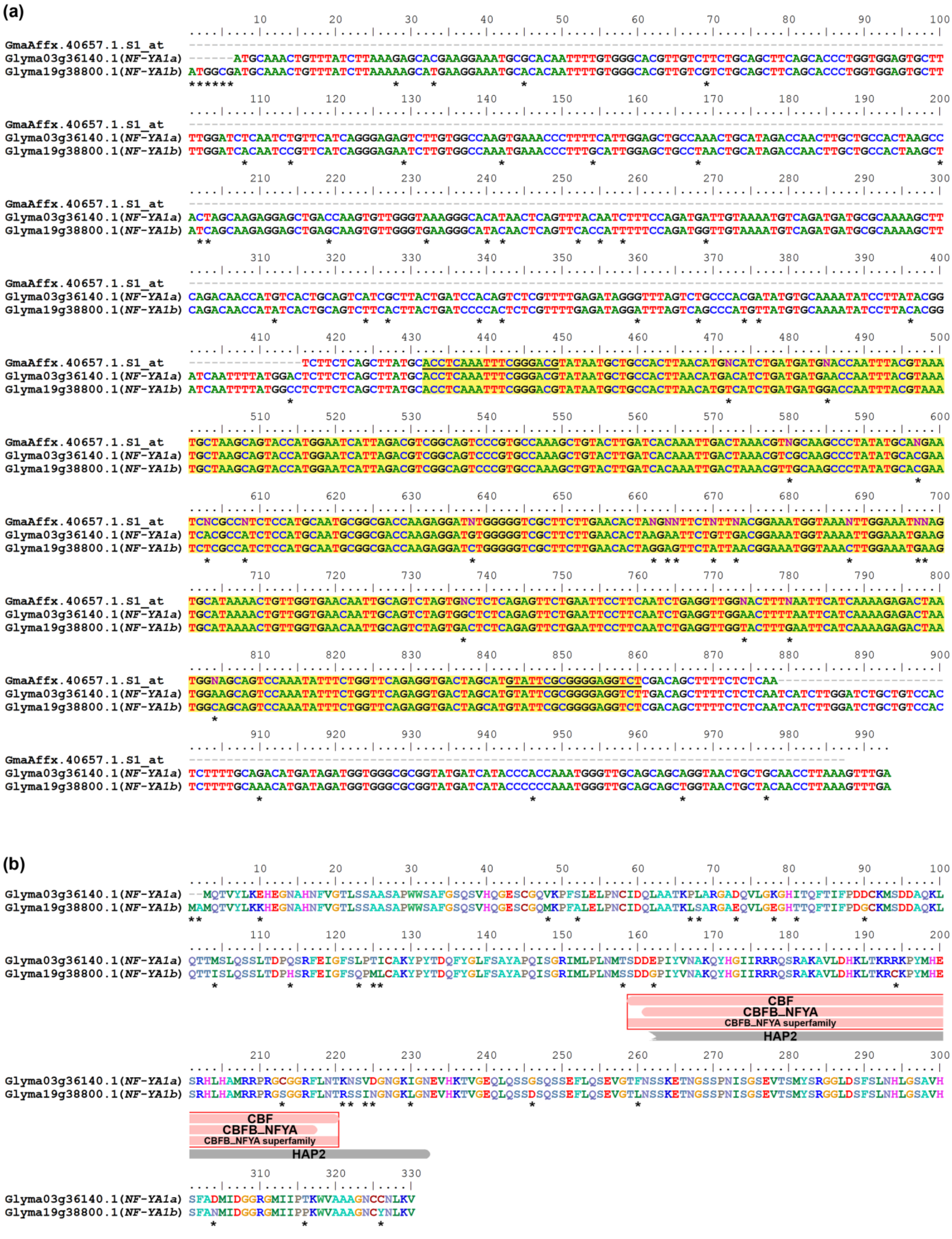

Figure S8. Sequence information of the putative *CCAAT-binding TF* genes targeted by GmaAffx.40657.1.S1_at.

(a) Nucleotide alignment of the target sequence of GmaAffx.40657.1.S1_at with the predicted coding sequences of Glyma03g36140 (*GmNF-YA1a*) and Glyma19g38800 (*GmNF-YA1b*). Note that the CDS of the current primary transcript Glyma19g38800.2 (Phytozome v9.0 [79]) contains 48 additional nucleotides at the 5'-end that are not included here. For creating the RNAi construct, the yellow-marked region was used. Primer binding sites for

amplification are underlined. The cloned sense and antisense arms were 100% identical to the Glyma03g36140 sequence.

(b) Amino acid alignment of NF-YA1a and NF-YA1b and conserved protein domains. To find conserved protein domains, the NCBI CD-search tool (http://www.ncbi.nlm.nih.gov/Structure/cdd/wrpsb.cgi) was used.

Both alignments were performed with the free software BioEdit (http://www.mbio.ncsu.edu/bioedit/bioedit.html). Asterisks indicate mismatches in the sequence alignments.

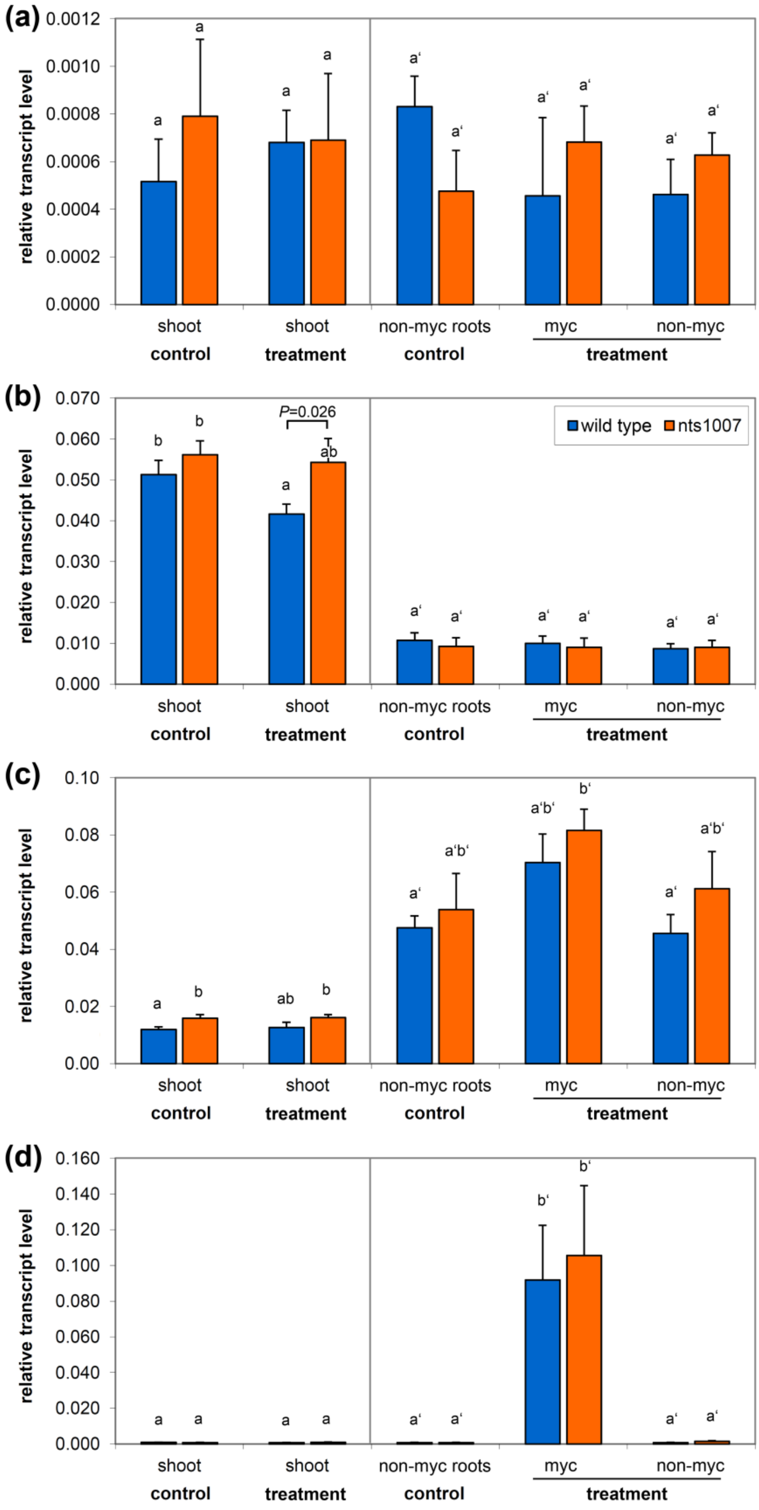

Figure S9. Affymetrix gene expression data of other putative *NF-Y* genes in soybean plants 19 days after inoculation.

(a) Gma.8502.1.S1_at, targeting Glyma20g00240 (putative *NF-YB*).

(b) Gma.12719.1.S1_at, targeting Glyma02g46970 (putative *NF-YB*).

(c) Gma.3174.1.S1_at, targeting Glyma09g07960 (putative *NF-YA*).

(d) GmaAffx.66953.1.S1_at, targeting Glyma12g34510 (putative *NF-YC*).

Affymetrix chip signals for putative *NF-Y* genes were set in relation to chip signals for GmaAffx.93644.1.S1_x_at targeting *GmSUBI-1* (see Figure S4m). Data are mean chip signal

+ SD of three biological replicates. For details see the Materials and methods section. Different letters indicate significant differences ($P \leq 0.05$, multiple Student's t-tests with Bonferroni correction performed separately for shoots and root-parts). The given P value in (a) derived from a pair-wise comparison. Data of all predicted *NF-Y* genes located on the Affymetrix GeneChip for soybean are listed in Table S3 in Additional file 2.

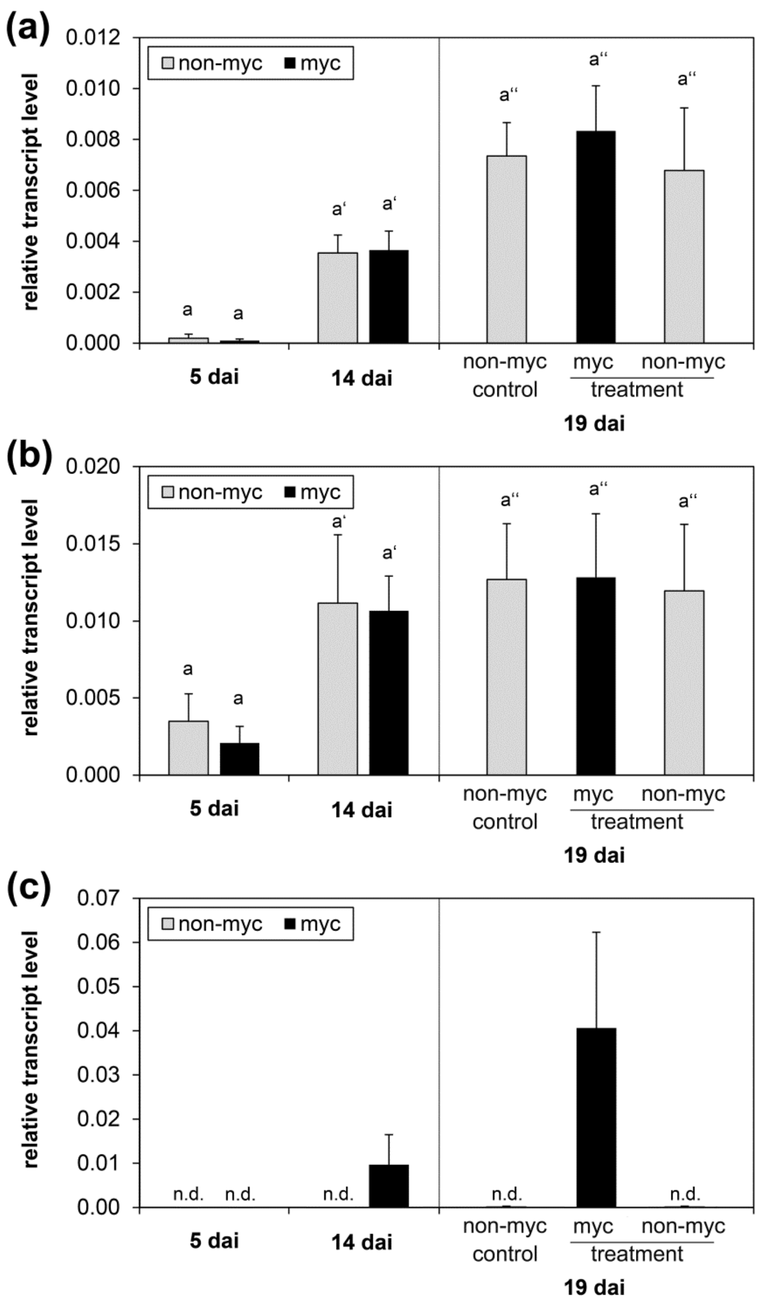

Figure S10. Transcript accumulation of the putative *NF-YA* genes Glyma10g10240 and Glyma02g35190 in root tissue upon *R. irregularis*-inoculation.

Relative Transcript levels of the *NF-YAs* Glyma10g10240 **(a)** and Glyma02g35190 **(b)** and of the AM fungal gene *RiBTub1* as marker for root colonization **(c)**. Plants harvested 5 and 14 days after inoculation (dai) were cultivated in a non-split-root system and were either inoculated with *R. irregularis* ('myc') or stayed non-inoculated ('non-myc') (n = 5-6). Plants harvested 19 days after inoculation are from two independent split-root experiments (total n = 9). Transcript levels were determined by RT-qPCR and set in relation to *GmSUBI-1*. Data are presented as means + SD. For each time-point, data of non-inoculated and inoculated root samples were compared by the Student's t-test with Bonferroni correction. Note that no significant (with $P \leq 0.05$) transcript regulation by AM was detected. However, both genes showed a developmentally regulated mRNA accumulation. n.d.: not detected.

Table S4: Sequences of primers used for RT-qPCR analysis and for creating the RNAi construct.

Target name	Forward primer (5'-3') [a]	Reverse primer (5'-3') [a]	Reference
Glyma15g15171	TGGGCGAAATAAAGGTGATGAA	GGCTCCGCCATTTTACAGATAGT	
Glyma18g17440	CCTGGCATTGATCATTTTCTC	CATCTTGTATTTCCAATAGACGTGA	
GmSIK1 (Glyma02g11150)	GGTGTGAGTTGCTACATCGATCA	TTACGAATTCCGCACTTGTATGTT	
Glyma17g09270	CCTGCGTTGACTGCATTGG	TGGCATTGGAACCTGACGTT	
Glyma10g35000	GACAAACCCGTTGGCACTCA	TTGGAAGCTGAACCCTTGGAT	
Glyma07g36986	CAGATGCAACCATGAATCCTTTAA	TGGAGGCAATAACAGCAGAAATT	
Glyma15g38010	AGGTTCTGCGCAACGCTCTA	CTCTTTGATGTCCCTGAGGTCCTT	
Glyma13g26960	GCAATGCGATAAAAGGGGTTGG	CTCTTTGATGTCCCTGAGGTCCTT	
GmNF-YA1 (Glyma03g36140+ Glyma19g38800)	TCAGCTTATGCACCTCAAATTTCG	TCAATTTGTGATCAAGTACAGCTTTGG	
GmNF-YA1a (Glyma03g36140)	AGCTTCGTTTCAGTTGCGTG	TACAATGAGCCAAGGATGGA	
GmNF-YA1b (Glyma19g38800)	AGCTTCTTTTGCGTGTGTTG	TACAATGAGCCAAGGATGGA	
Glyma10g10240	ACATGGTATTATCACGCCCACTA	AGAATCACACCTCCAGAACGAA	
Glyma02g35190	GCAGACATGATAGGTAGTGAGCA	GGCACGCCTCCAAAACAAA	
Glyma13g08720	TCATTTTCGCGGGTTTAGTC	GCTTGCTTCACGTTTCCTTC	[86]
Glyma14g28780	ATGTTTAACTGTGGGCGGCG	CCCTATTATTGGGCGTCGGT	[86]
Glyma14g36650	GGACTCCCGAATGAATGCTA	AGCTGCAGTCAACTCCCCTA	[86]
RiBTub1	CCAACTTATGGCGATCTCAACA	AAGACGTGGAAAAGGCACCA	[56]
GmSUBI-1	TGCAGATTTTTGTGAAGACTCTTACG	CCCTCCTTGTCCTGAATCTTAGC	
RNAi sense-arm	ggatccACCTCAAATTTCGGGACGTA	actagtAGACCTCCCCGCGAATAC	
RNAi antisense-arm	*ggcggcgc*atttaaatACCTCAAATTTCGGGACG	*ttatat*ggcgcgccAGACCTCCCCGCGAATAC	

[a]uppercase letters indicate gene-specific sequences, lowercase letters indicate attached restriction sites, italic letters indicated bases attached for improved GC content and stability of primer
